# Logistic regression with sparse common and distinctive covariates

**DOI:** 10.3758/s13428-022-02011-2

**Published:** 2023-02-13

**Authors:** S. Park, E. Ceulemans, K. Van Deun

**Affiliations:** 1 Tilburg University, Tilburg, Netherlands; 2 KU Leuven, Leuven, Belgium

**Keywords:** Multiblock data, Principal covariates regression, Common and distinctive processes, Data integration, Classification, Logistic regression

## Abstract

Having large sets of predictor variables from multiple sources concerning the same individuals is becoming increasingly common in behavioral research. On top of the variable selection problem, predicting a categorical outcome using such data gives rise to an additional challenge of identifying the processes at play underneath the predictors. These processes are of particular interest in the setting of multi-source data because they can either be associated individually with a single data source or jointly with multiple sources. Although many methods have addressed the classification problem in high dimensionality, the additional challenge of distinguishing such underlying predictor processes from multi-source data has not received sufficient attention. To this end, we propose the method of Sparse Common and Distinctive Covariates Logistic Regression (SCD-Cov-logR). The method is a multi-source extension of principal covariates regression that combines with generalized linear modeling framework to allow classification of a categorical outcome. In a simulation study, SCD-Cov-logR resulted in outperformance compared to related methods commonly used in behavioral sciences. We also demonstrate the practical usage of the method under an empirical dataset.

## Introduction

In behavioral research, it is often of interest to classify subjects, e.g., by constructing a logistic regression model. For example, in mental health research, scores on various tests are used to classify subjects into having versus not having a disorder such as alcoholism (Babor, Higgins-Biddle, Saunders, & Monteiro, 2001), dementia (Mioshi, Dawson, Mitchell, Arnold, & Hodges, 2006), and eating disorders (Hill, Reid, Morgan, & Lacey, 2010; Botella, Huang, & Suero, 2015). By constructing a classification model, the factors predicting class membership can be investigated. For example, Barnes et al., (2009) studied the importance of various measures such as genotype, fMRI, and cognitive tests in predicting dementia among older adults through logistic regression. As a result, a risk index that stratifies older adults into different risk groups depending on their scores on certain risk factors was put forward.Many studies in behavioral sciences of today involve datasets comprised of multiple blocks of predictor variables obtained for the same individuals, with each block of variables originating from different measurement instruments. Examples of such blocks include demographic data, social media, genetic profiling, and questionnaires. These joint datasets are referred to as multiblock data (more details on the conceptual framework are given in Van Mechelen and Smilde, 2010). A unique feature of multiblock data is that they can reveal two different kinds of sources of interindividual variation; those that concern single individual data blocks and those that jointly encompass multiple blocks. These sources of variation are referred to as distinctive and common, respectively, and they are used to reveal the processes underlying the emergence of particular conditions. To explain more concretely, let us consider a block of genotype data and another block of self-reported health behavior data collected from two groups of children; ADHD-diagnosed and healthy. Studying the onset of ADHD by adopting this multiblock dataset, processes that only underlies the genotype data may be found. For example, a dopaminergic pathway involving dopamine transporter gene (DAT1) and a serotonergic pathway incorporating serotonin transporter gene (5HTTT) have been reported to play a role in ADHD (Gizer, Ficks, & Waldman, 2009). These biological pathways would be considered as distinctive processes as they entail only the genotype data block. On the other hand, the multiblock data could also reveal a process that involves both blocks of genotype and health behavior. Kahn, Khoury, Nichols, and Lanphear (2003) found the combination of maternal prenatal smoking with a DAT1 genotype leading to ADHD, while in another study, maternal stress during pregnancy together with dopamine receptor 4 gene (DRD4) were associated with severity of ADHD symptoms (Grizenko et al., 2012). Such cases of gene–environment interplay are examples of common processes as they involve multiple data blocks.

Methods based on PCA have been actively proposed to disentangle the common and distinctive processes from multiblock data, but without considering the prediction problem of an outcome variable (e.g., simultaneous component analysis with distinctive and common components, DISCO-SCA; Schouteden et al.,, 2013). As multiblock datasets are often characterized by a large number of variables, these PCA based methods have been further extended. The presence of many variables complicates the interpretation of the components derived by SCA as they are associated with a large set of variables. The introduction of sparseness penalties—limiting the number of variables associated with a component—yields interpretable components that represent common and distinctive processes (e.g., sparse common and distinctive SCA (SCaDS); de Schipper & Van Deun, 2018).

Recently, a method that identifies common and distinctive processes from a multiblock dataset in the context of a regression problem for a continuous outcome has been proposed (Sparse Common and Distinctive Covariates Regression (SCD-CovR); Park et al.,, 2020). The method is an extension of principal covariates regression (PCovR) which finds summary variables that explain variance in both predictors and outcome by combining PCA and linear regression (De Jong & Kiers, 1992). SCD-CovR incorporates SCaDS into the PCovR framework to obtain sparse common and distinctive predictor processes. In order to address the classification problem, the current paper extends the SCD-CovR method to logistic regression; this means that here we develop sparse common and distinctive covariates logistic regression method (SCD-Cov-logR). SCD-Cov-logR reveals the common and distinctive predictor processes that play a role in classification of the outcome and does so in an interpretable/insightful way by relying on sparse representations.

The paper is arranged as follows. First, we provide the methodological background and mathematical details of SCD-Cov-logR. Then, the results from simulation studies that comparatively demonstrate the performance of SCD-Cov-logR against an existing method with a similar set of objectives are presented. After further illustration of the current method on an empirical multiblock dataset, the paper is concluded by formulating some limitations and directions for future research. The implementation of SCD-Cov-logR was done in R and Rcpp, which can be found on GitHub: https://github.com/soogs/SCD-Cov-logR, along with the code used to generate the results reported in the paper.

## Methods

### Notation

The following notation is used throughout the paper: scalars, vectors and matrices are denoted by italic lowercase, bold lowercase and bold uppercase letters respectively. Transposing is indicated by the superscript ^*T*^. Lowercase subscripts running from 1 to corresponding uppercase letters denote indexing: $i \in \lbrace 1,2, \dots , I \rbrace $. Subscript _*C*_ indicates concatenation of multiple data blocks, while superscripts ^(*X*)^, ^(*y*)^ and ^(*g*)^ highlight affiliation with predictor, continuous outcome and binary outcome variables, respectively. To denote estimates, a $\hat {}$ over the symbol denoting the population parameter is used (i.e., $\hat {\mathbf {b}}$ is the estimated logistic regression coefficients). **X** refers to a matrix containing the standardized scores of *J* predictors corresponding to *I* observation units (that is, each column has mean zero and variance equal to one). In the context of multiple predictor blocks, **X**_*k*_ (with size *I* × *J*_*k*_) indicates a *k* th predictor block matrix with its predictors column-scaled and standardized; with *k* ∈{1,2,…,*K*}. $\mathbf {X}_{C}= \left [ \mathbf {X}_{1}, \ldots , \mathbf {X}_{K} \right ]$ (of size $I \times {\sum }^{K}_{k = 1} J_{k}$) denotes the supermatrix that concatenates the predictor blocks. **g** indicates a dummy vector of size *I* containing the scores on the binary outcome variable, while **y** is a vector of size *I* of a continuous outcome. In the context of an outcome variable with multiple categories, **G** (with size *I* × *M*) refers to a dummy matrix for the categorical outcome with *M* total categories. For the *i* th observation unit, *g*_*im*_ = 1 if the response is in the *m* th category and *g*_*im*_ = 0 otherwise. Lastly, **I**_*a*_ denotes a *a* × *a* identity matrix where the subscript *a* indicates the size of the matrix.

### Model and objective function

SCD-Cov-logR is a classification method for a categorical outcome. The method is particularly suitable when multiple large blocks of predictor variables are available as it allows to take the block structure into account and to limit the number of variables contributing to the predictive processes. SCD-Cov-logR constructs two types of summary covariates: distinctive covariates based on a linear combination of the predictor variables of one single data block and common covariates that combine variables of multiple data blocks. Identification of different types of predictor processes helps understanding of processes that play important roles in the classification of the outcome. To further facilitate the interpretation of these processes, SCD-Cov-logR introduces regularization penalties to select a subset of the predictor variables in constructing the common and distinctive covariates. Taken together, an effective classification method results where common and distinctive predictor processes are identified in a sparse and therefore interpretable manner; the method is also flexible in the sense that it includes several other methods as a special case such as logistic regression and PCovR for categorical outcomes. We start with a brief description of the building blocks, namely logistic regression and PCovR, before moving onto SCD-Cov-logR. While the current method allows classification of both binary and multiclass outcome variables via logistic regression, we focus on binary logistic regression in the following subsections in describing our method. The multiclass classification via multinomial logistic regression will be discussed thereafter, as it is a straightforward extension of the binary problem.

#### Logistic regression

Logistic regression assumes that the log-odds (logit) of the binary outcome are linearly dependent on the predictor variables. Let **x**_*i*_ be the vector of predictor scores for subject *i* and *g*_*i*_ the score on the outcome (either 0 or 1). The log-odds for subject *i* is modeled by:

1 $$  \log \left( \frac{p(g_{i} = 1)}{1 - p(g_{i} = 1)} \right) = \mathbf{x}_{i}^{T} \mathbf{b} + b_{0} $$

where *p*(*g*_*i*_ = 1) denotes the probability that the *i* th subject would fall under the category represented by a 1. The vector **b** indicates the logistic regression weights and the scalar *b*_0_ the intercept. From this model, it follows that

2 $$ \begin{array}{@{}rcl@{}} p(g_{i} = 1)& = & \frac{1}{1 + e^{-(\mathbf{x}_{i}^{T} \mathbf{b} + b_{0})}} \\ p(g_{i} = 0)& = & 1 - p(g_{i} = 1), \end{array} $$

which can be used to set up the likelihood equation. The estimates of the logistic regression parameters can then be obtained by maximizing the log-likelihood or minimizing the negative log-likelihood; here, the latter will be used for integration with the PCovR objective. The following negative log-likelihood is minimized:

3 $$  L(\mathbf{b}, b_{0}) = -{\sum\limits_{i}^{I}} (g_{i} (b_{0} + {\mathbf{x}}_{i}^{T} \mathbf{b}) - \log(1 + e^{(b_{0} + \mathbf{x}_{i}^{T} \mathbf{b})})). $$

Typically, the minimum of this function is found via a numerical procedure as it has no closed form. A popular approach is the Newton–Raphson method for finding the root of the first derivative which amounts to iteratively reweighted least squares. It boils down to formulating local quadratic approximations of the negative log-likelihood in an iterative scheme that, after initialization, uses the minimum of the quadratic approximation for updating in the next iteration.

#### PCovR

In a setting with a large set of predictor variables, the ordinary (least-squares) approach to linear regression involves several drawbacks. It is difficult to interpret the large set of regression coefficients corresponding to each of the predictors. Also, in the case of multicollinearity (highly correlated predictors), the estimates are instable. When the number of predictors exceeds the number of observations (high-dimensionality), the method has no unique solution. In order to alleviate these difficulties, Principal Covariates Regression (PCovR; De Jong & Kiers 1992) was put forward by combining PCA with linear regression. PCovR introduces summary variables, the so-called ‘principal covariates’, in modeling the predictor and outcome variables. The covariates summarize the predictors by a linear combination of the original variables that is obtained in such a way that they account for variation in both predictor and outcome variables. Regression coefficients are found for these limited number of covariates instead of for each of the original predictor variables, resolving the challenges of finding a unique and stable regression model in the setting of a large number of predictors. Since the covariates summarize the predictors, they can be understood to represent the predictor processes behind the outcome. Let *R* be the pre-specified number of covariates to be derived. PCovR then assumes the following models for the predictor and outcome variables:

4 $$ \begin{array}{@{}rcl@{}} \mathbf{y} & = & \mathbf{X} \mathbf{W} \mathbf{p}^{(y)} + \mathbf{e}^{(y)} \\ \mathbf{X} & = & \mathbf{X} \mathbf{W} (\mathbf{P}^{(X)})^{T} + \mathbf{E}^{(X)}. \end{array} $$

Both the models for the outcome **y** and for the predictor variables **X** rely on the same summary predictor scores **XW** with **W** referring to the weights matrix of size *J* × *R*. The weights prescribe the linear combination of the predictors to compose the principal covariates (namely, **T** = **XW**). The first line of Eq. 4 shows the model underlying the outcome; in that model **p**^(*y*)^ indicates a vector of *R* regression coefficients while **e**^(*y*)^ denotes the residuals pertaining to the outcome. The second line of Eq. 4 gives the model for the predictors. **P**^(*X*)^ indicates the loadings matrix of size *J* × *R*. Similar to the regression coefficients **p**^(*y*)^ for the outcome variable in the first line, the loadings matrix linearly combine the covariates to reconstruct back the predictors. It can be seen as regression coefficients obtained from regressing the predictor variables on the principal covariates. Note that this model formulation also underlies the methods of principal components regression (PCR; see Jolliffe, 1982) and partial least squares (PLS; Wold, 1982; Wold et al.,, 1983).

The aim of PCovR to find covariates that effectively reconstruct **X** and simultaneously predict **y** is expressed by the following joint loss function (De Jong & Kiers, 1992):

5 $$ \begin{array}{@{}rcl@{}} L(\mathbf{W}, \mathbf{P}^{(X)}, \mathbf{p}^{(y)}) &=& \alpha \frac{\left\| \mathbf{y} - \mathbf{X} \mathbf{W} \mathbf{p}^{(y)} \right\|_{2}^{2}}{\left\| \mathbf{y} \right\|_{2}^{2}} \\&&+ (1 - \alpha) \frac{\left\| \mathbf{X} - \mathbf{X} \mathbf{W} (\mathbf{P}^{(X)})^{T} \right\|_{2}^{2}}{\left\| \mathbf{X} \right\|_{2}^{2}}, \end{array} $$

with 0 ≤ *α* ≤ 1, a known constant which specifies the balance between fitting the outcome and the predictors. With *α* set at 0, the method is the same as PCR where the outcome variable is regressed on the principal components found by PCA. On the other hand, with *α* = 1, the method is equivalent to linear regression.^1^ The solution of Eq. 5 is not identifiable without imposing constraints. Therefore, the covariates are often constrained to be orthonormal ($\mathbf {T}^{T} \mathbf {T} = \mathbf {I}_{R}$) to identify the solution (De Jong & Kiers, 1992).

The principal covariates in the PCovR model are used to represent the processes that underlie both the predictor and outcome variables. Therefore, it is important to interpret the derived covariates to understand the nature of these processes. There are two ways of interpreting the covariates. Firstly, the loadings matrix **P**^(*X*)^ can be studied. When the principal covariates are scaled to variance equal to one ($\mathbf {T}^{T} \mathbf {T} = I \mathbf {I}_{R}$) and the predictor variables have been centered and scaled to variance equal to one, the loadings are equal to the correlation between the principal covariates and the predictor variables. Therefore, **P**^(*X*)^ can be conveniently interpreted in two ways; regression coefficients that reconstruct the predictors (namely, **T**(**P**^(*X*)^)^*T*^ = (**XW**)(**P**^(*X*)^)^*T*^ = **X**) and covariate-predictor correlations. The loadings derived within PCA are also commonly studied in the same manner. On the other hand, the second way to understand the covariates is by observing the weights matrix **W**. The weights are used in combining the predictors to construct the covariates, and therefore they describe the composition of the covariates. They also play an important role in applying the model to new data, in the context of prediction for new observations, as they are used to transform the new predictor variables to covariate scores. Studying the loadings or the weights are both valid ways to understand the nature of the covariates and the two estimates can both be inspected in a complementary manner. However, if one of the estimates should be chosen for inspection, the choice should depend on the research aim of interest; loadings reflect the strength of association of the predictor variables with the principal covariates while weights prescribe how the covariates are constructed. We refer to Guerra-Urzola, Van Deun, Vera, and Sijtsma (2021) for a thorough discussion of the issue of loadings versus weights in the context of sparse PCA.

#### SCD-Cov-logR

Here, we propose a method for binary classification that is suitable for multiblock data where several blocks of predictor variables are available: besides the fact that the method can handle many predictors or even high-dimensional data, it yields particular insight in the data by revealing common and distinctive predictor processes in a sparse and therefore interpretable manner.

##### Model

We make use of a model formulation that integrates the logistic regression and PCovR models in Eqs. 2 and 4. More specifically, the model for the outcome variable is adapted. Let the vector **x**_*C*__*i*_ denote the *i* th row of the supermatix **X**_*C*_ resulting from the concatenation of the predictor blocks and let **W**_*C*_ of size ${\sum }^{K}_{k = 1} J_{k} \times R$ denote the corresponding weights matrix, then the log-odds of the binary outcome can be modeled by the principal covariates as follows:

6 $$ \begin{array}{@{}rcl@{}} \log \left( \frac{p(g_{i} = 1)}{1 - p(g_{i} = 1)} \right)& = & {\mathbf{x}_{C}}_{i}^{T} \mathbf{W}_{C} \mathbf{p}^{(g)} + p^{(g)}_{0} \\ {\mathbf{x}_{C}}_{i} & = & \left[ {\mathbf{x}_{C}}_{i}^{T} \mathbf{W}_{C} (\mathbf{P}_{C}^{(X)})^{T} \right]^{T} + \mathbf{e}_{i}^{(X)}, \end{array} $$

where **p**^(*g*)^ in the first line of the equation denotes the vector of *R* regression coefficients and $p^{(g)}_{0}$ the intercept. As in the PCovR model (Eq. 4), the weights matrix dictates the composition of the covariates (**T**_*C*_ = **X**_*C*_**W**_*C*_). In the second line, $\mathbf {P}_{C}^{(X)}$ indicates the loadings matrix of size ${\sum }^{K}_{k = 1} J_{k} \times R$. They recover the predictor variables from the covariates, as done in the PCovR model. Therefore, the covariates in this model explain both the variance of predictor variables and the log-odds of the binary outcome variable.

The model in Eq. 6 includes all predictor variables in constructing the principal covariates while often it is of interest to find the subset of variables that are relevant for the predictor processes represented by the principal covariates. Hence, our proposed model is subject to a sparsity inducing penalty that limits the number of predictor variables contributing to the covariates. SCD-Cov-logR therefore imposes the sparsity on the weights, as we are interested in finding a subset of predictors that together make up the predictor processes. In this way, understanding the covariates becomes much easier as they are based on a smaller subset of predictors.

To understand the composition of the covariates not only at the level of the individual variables but also at the level of the blocks, sparsity is imposed in two ways: On the one hand at the level of the blocks (blockwise sparsity) and, on the other hand, at the level of the individual variables (elementwise sparsity). Blockwise sparsity refers to forcing the weights corresponding to an entire set of predictors in a data block to zero. By doing so, distinctive covariates which are only comprised of predictors from a single data block can be obtained. If more than one predictor blocks but not all make up a covariate, that would be referred to as a locally common covariate, as opposed to a globally common covariate where all of the predictor blocks are involved in deriving the covariate (Måge, Smilde, & Van der Kloet, 2019). Elementwise sparsity indicates dropping individual predictors out of the model. Combining these two types of sparsity encouraged at different levels, only a subset of predictors within the blocks that are chosen by blockwise sparsity would be left in the model to make up a covariate. Common and distinctive covariates that are comprised of a small interpretable subset of predictors can therefore be found to represent the underlying predictor processes.

##### Objective function

In setting up the objective function of SCD-Cov-logR, the objectives for logistic regression and PCovR are combined. As discussed, for a binary outcome the log-odds are regressed on the covariates. Hence, the squared error pertaining to the outcome (the left term in Eq. 5) is replaced by a negative log-likelihood function based on the PCovR logistic regression model (first line in Eq. 6). Furthermore, the two types of sparsity on the weights **W**_*C*_ are accomplished by imposing two different penalties. We employ the group lasso penalty (Yuan & Lin, 2006) which shrinks and sparsifies the weights at the block level, and the lasso penalty (Tibshirani, 1996) that does the same but for individual weights. This combination of penalties is also known as the sparse group lasso (Friedman, Hastie, & Tibshirani, 2010a; Simon, Friedman, Hastie, & Tibshirani, 2013). The objective of SCD-Cov-logR is to minimize the following loss function,

7 $$ \begin{array}{@{}rcl@{}} &&\!L(\mathbf{W}_{C}, \mathbf{P}^{(X)}_{C}, \mathbf{p}^{(g)}, p^{(g)}_{0})\\ &\! = &\frac{\alpha}{l_{0}} \left\lbrack\!-{\sum_{i}^{I}} (g_{i} (p^{(g)}_{0} + {\mathbf{x}_{C}}_{i}^{T} \mathbf{W}_{C} \mathbf{p}^{(g)}) - \log(1 + e^{(p^{(g)}_{0} + {\mathbf{x}_{C}}_{i}^{T} \mathbf{W}_{C} \mathbf{p}^{(g)})}))\!\right\rbrack\\ && \!+ \frac{1 - \alpha}{\left\| \mathbf{X}_{C} \right\|^{2}_{2}} {\sum_{i}^{I}} \left\| {\mathbf{x}_{C}}_{i}^{T} - {\mathbf{x}_{C}}_{i}^{T} \mathbf{W}_{C} (\mathbf{P}_{C}^{(X)})^{T} \right\|^{2}_{2} \\ && \!+ {\sum\limits_{r}^{R}} {\lambda_{L}}_{r} \left| {\mathbf{w}_{C}}_{r} \right|_{1} + {\sum\limits_{r}^{R}} {\sum\limits_{k}^{K}} {\lambda_{G}}_{r} \sqrt{J_{k}} \left\| \mathbf{w}^{(k)}_{r} \right\|_{2} + \lambda_{R} \left\| \mathbf{p}^{(g)} \right\|^{2}_{2} \end{array} $$

where the loadings associated with the predictors $\mathbf {P}_{C}^{(X)}$ are constrained to be column-orthogonal ($(\mathbf {P}_{C}^{(X)})^{T} \mathbf {P}_{C}^{(X)} = \mathbf {I}_{R}$) in order to identify the solution (and to avoid an ill-posed problem resulting in ever-decreasing weights compensated by ever-increasing loadings). *l*_0_ refers to the negative log-likelihood of the null model fitted without any predictors $l_{0} = -{{\sum }_{i}^{I}} \left (g_{i} \log (\bar {p}) + (1-g_{i}) \log (1 - \bar {p}) \right )$, where $\bar {p} = \frac {1}{I} {{\sum }_{i}^{I}} g_{i}$ is the proportion of observations in the first category. The terms with *λ*_*G*__*r*_ and *λ*_*L*__*r*_ refer to the group lasso and the lasso penalties corresponding to the *r* th covariate. $\mathbf {w}^{(k)}_{r}$ indicates the weights corresponding to the covariate *r* and the predictor block *k*. The last term denotes the ridge penalty imposed on the regression coefficients **p**^(*g*)^ to prevent divergence occurring due to covariates being correlated.

The first term of the loss function represents the negative log-likelihood function based on Eq. 6. It is in the same format as the negative log-likelihood function commonly used for logistic regression, except that it has been adapted according to the multiblock PCovR model structure. This term is divided by the log-likelihood of the null model^2^*l*_0_, while the second term of sum of squared predictor errors is divided by the total sum of squared predictor scores. The two types of losses are therefore placed within a comparable scale between 0 and 1. With respect to the penalties on the weights, it can be seen that the group lasso penalty $\left \| \cdot \right \|_{2}$ concerns a group of weights connecting the predictors in the *k* th predictor block with the *r* th covariate, while the lasso penalty $\left | \cdot \right |_{1}$ is imposed on all of the ${\sum }^{K}_{k = 1} J_{k}$ individual weights corresponding to *r* th covariate. The two penalties together make up the sparse group lasso.

It is possible to re-express the objective function by scaling the *α* parameter such that it already takes account of the negative log-likelihood of the null model *l*_0_ and the sum of squared predictor scores $\left \| \mathbf {X}_{C} \right \|^{2}_{2}$. The scaled weighting parameter *β* is defined by:

8 $$  \beta = \frac{\alpha \left\| \mathbf{X}_{C} \right\|^{2}_{2}}{\alpha \left\| \mathbf{X}_{C} \right\|^{2}_{2} + (1-\alpha) l_{0} } $$

*β* can then replace $\frac {\alpha }{l_{0}}$ in the objective function (7) while (1 − *β*) replaces $\frac {(1-\alpha )}{\left \| \mathbf {X}_{C} \right \|^{2}_{2}}$, leading to a different expression of the same objective. Such rescaling of the weighting parameter has been shown in Vervloet, Van Deun, Van den Noortgate, and Ceulemans (2013).

##### Relation to existing methods

Several existing methods rely on objective functions that are similar to the objective introduced here in Eq. 7. A method called Sparse Principal Component Regression (SPCR; Kawano et al.,, 2018) has been proposed and combined with generalized linear modeling. SPCR and SCD-Cov-logR are characterized by similar objective functions; our method can be viewed as an extension of SPCR for the setting of multiple predictor blocks. Likewise, several other methods can be seen as a special case of the objective function in Eq. 7. First, if the balancing parameter *α* is fixed at zero, common and distinctive sparse covariates would be found only optimizing the fit to the predictor variables. This solution would be equivalent to that of SCaDS (de Schipper & Van Deun, 2018), which finds common and distinctive sparse components from multiblock data. For this reason, and also because the algorithm for SCD-Cov-logR is infeasible when *α* is equal to exactly zero, we rely on SCaDS to find the solutions when *α* = 0. Second, if the negative log-likelihood term is replaced by squared error pertaining to a continuous outcome ($ \left \| \mathbf {y} - \mathbf {X}_{C} \mathbf {W}_{C} \mathbf {p}^{(y)} \right \|_{2}^{2} / \left \| \mathbf {y} \right \|_{2}^{2}$), the objective function becomes that of SCD-CovR (Park, Ceulemans, & Van Deun, 2020), which shares the same aims as SCD-Cov-logR except it targets a continuous outcome. Third, starting from the SCD-CovR formulation, if the group lasso parameter is fixed at zero and only a single block of predictors are employed, the problem boils down to SPCovR (Van Deun, Crompvoets, & Ceulemans, 2018) which finds sparse covariates. As these methods serve as the basis for the current SCD-Cov-logR, further details of these directly related methods are provided in Appendix A. Finally, fixing the lasso and group lasso parameters at zero such that weights are found without sparsity, the problem can be seen as an extension to PCovR to account for a binary classification problem.

##### Algorithm

The minimizing solution of Eq. 7 can be found by an alternating procedure where the loadings $\mathbf {P}^{(X)}_{C}$ and the regression coefficients **p**^(*g*)^ and $p^{(g)}_{0}$ are solved for conditional upon fixed values for the weights **W**_*C*_ and vice versa. Such an alternating approach has been effective for SCaDS, SCD-CovR and SPCovR. To treat the minimization of Eq. 7 which is complicated by the negative log-likelihood term, we make use of a local quadratic approximation, similar to the iteratively reweighted least squares approach that is usually taken to solve the logistic regression problem (Friedman, Hastie, & Tibshirani, 2010b). The alternating routine continues until the algorithm converges to a stationary point, usually a local minimum. Since the iteratively reweighted least squares procedure is known to sometimes lead to divergence, we also employ the maximum number of iteration of 5000 as another form of stopping criterion. As the objective function in Eq. 7 is not a convex problem, it is subject to local minima. We recommend using multiple random starting values, along with rational starting values based on PCovR (administered by treating the binary outcome as a continuous variable). Furthermore, employing multiple starting values is particularly important because the estimation of **W**_*C*_ is often a high dimensional regression problem prone to instable estimates (Jia & Yu, 2010; Guerra-Urzola, Van Deun, Vera, & Sijtsma, 2021), meaning that different starting values may result in different estimates. The sparse group lasso problem for **W**_*C*_ is treated via coordinate descent (Friedman et al., 2010a), while closed-form solutions exist for the conditional updates of $\mathbf {P}_{C}^{(X)}$, **p**^(*g*)^ and $p^{(g)}_{0}$. Further details on the algorithm for minimizing the objective function can be found in the Appendix B, including the schematic outline of the algorithm and the derivation of solutions to the conditional updates (Appendices C and D).

#### Multiclass classification

Our method can be slightly adapted to address a classification problem in the presence of more than two categories. The method is posed in the same manner as the binary problem, except it relies on multinomial logistic regression. The logit model in Eq. 6 is generalized to a ‘baseline-category logit model’ (Agresti, 2003) which is a common approach to extend logistic regression to a multiclass problem. Let *p*(*g*_*im*_ = 1) and *p*(*g*_*iM*_ = 1) denote the probability that subject *i* would fall under the category *m* and the last category *M*, respectively. Treating the last category as the baseline, the log-odds of the *i* th observation being in category *m* as opposed to being in the baseline category is modeled:

9 $$ \begin{array}{@{}rcl@{}} \log \left( \frac{p(g_{im} = 1)}{p(g_{iM} = 1)} \right)& = & {\mathbf{x}_{C}}_{i}^{T} \mathbf{W}_{C} \mathbf{p}_{m}^{(g)} + {{p_{0}}^{(g)}_{m}},\\&& \text{ for } m = 1, \ldots, M-1 \\ {\mathbf{x}_{C}}_{i}& = & \left[ {\mathbf{x}_{C}}_{i}^{T} \mathbf{W}_{C} (\mathbf{P}_{C}^{(X)})^{T} \right]^{T} + \mathbf{e}_{i}^{(X)}, \end{array} $$

where $\mathbf {p}_{m}^{(g)}$ and ${{p_{0}}^{(g)}_{m}}$ refer to the regression coefficients and the intercept that correspond to category *m*. By calculating *M* − 1 sets of the regression coefficients, the log-odds of any pairs of response categories can be determined. As for the objective function, the negative log-likelihood function based on the baseline-category logit model replaces the negative log-likelihood concerning the binary classification provided in Eq. 7:

10 $$ \begin{array}{@{}rcl@{}} && L(\mathbf{W}_{C}, \mathbf{P}^{(X)}_{C}, \mathbf{p}_{m}^{(g)}, {{p_{0}}^{(g)}_{m}}) \\ & =& {\frac{\alpha}{l_{0}}} \left\lbrack - {\sum\limits_{i}^{I}} \left\{ \sum\limits_{m}^{M-1} g_{im} ({{p_{0}}^{(g)}_{m}} + {\mathbf{x}_{C}}_{i}^{T} \mathbf{W}_{C} \mathbf{p}_{m}^{(g)}) \right.\right.\\&&\left.\left.- \log(1 + \sum\limits_{m}^{M-1} e^{({{p_{0}}^{(g)}_{m}} + {\mathbf{x}_{C}}_{i}^{T} \mathbf{W}_{C} \mathbf{p}_{m}^{(g)})}) \right\} \right\rbrack\\ && + {\frac{1 - \alpha}{\left\|\mathbf{X}_{C} \right\|^{2}_{2}}} {\sum\limits_{i}^{I}} \left\| {\mathbf{x}_{C}}_{i}^{T} - {\mathbf{x}_{C}}_{i}^{T} \mathbf{W}_{C} (\mathbf{P}_{C}^{(X)})^{T} \right\|^{2}_{2} \\ && + {\sum\limits_{r}^{R}} {\lambda_{L}}_{r} \left| {\mathbf{w}_{C}}_{r} \right|_{1} + {\sum\limits_{r}^{R}} {\sum\limits_{k}^{K}} {\lambda_{G}}_{r} \sqrt{J_{k}} \left\| \mathbf{w}^{(k)}_{r} \right\|_{2} \\&&+ \lambda_{R} \left\| \mathbf{p}^{(g)} \right\|^{2}_{2} \end{array} $$

where the loadings $\mathbf {P}_{C}^{(X)}$ are constrained to be column-orthogonal ($(\mathbf {P}_{C}^{(X)})^{T} \mathbf {P}_{C}^{(X)} = \mathbf {I}_{R}$) as done for the binary problem (Eq. 7). Other quantities and penalty terms are also defined the same. *l*_0_ here refers to the negative log-likelihood of the null model $l_{0} = -{{\sum }_{i}^{I}}\left \lbrack {\sum }_{m}^{M-1} g_{im} \log (\bar {p}_{m}) +\right .$ $\left . g_{iM} \log (\bar {p}_{M}\vphantom {{\sum }_{i}^{I}}) \right \rbrack $ where $\bar {p}_{m} = \frac {1}{I} {{\sum }_{i}^{I}} g_{im}$ is the proportion of observations in the *m* th category. Hence, the negative log-likelihood and the sum of squared errors are also scaled in this objective function. The weighting parameter *α* can be rescaled to *β* in the same manner as for the binary classification problem (see Eq. 8). Furthermore, note that both the model (Eq. 9) and the objective function (Eq. 10) become equal to those of the binary problem (Eqs. 6 and 7) when the total number of categories *M* are set at two. To find the minimizing solution of Eq. 10, an alternating algorithm very similar to that for the binary problem is employed. The only difference is that the negative log-likelihood term with multiple categories is treated with partial quadratic approximation with respect to the category *m* where only $\mathbf {p}_{m}^{(g)}$ and ${{p_{0}}^{(g)}_{m}}$ are allowed to vary at a time. This partial quadratic approximation has been used for treating a penalized multinomial logistic regression problem (Friedman et al., 2010b). Details on the algorithm are provided in the Appendix E.

### Toy example

In order to provide a clearer picture of the goals that the method targets and the estimates it provides, we showcase the method on a toy example dataset for a binary classification problem in this section. We generated the dataset according to one of the conditions of the simulation study which follows later. The dataset is composed of two data blocks and its underlying model assumes three covariates. Two of these covariates represent processes that are distinctive to the first and the second data blocks, respectively, while the third covariate is a common process, affiliated with both data blocks. In addition, the model was defined such that the covariate distinctive to the second block is not relevant in the classification of the outcome variable. Each of the two data blocks consists of 15 predictors concerning the same set of 100 observation units. There is one binary outcome variable. Details of the data generation setup can be found in the simulation study section.

A few technicalities come with the application of the SCD-Cov-logR to data. First, it is important to note that the solution is influenced by several tuning parameters that need to be fine-tuned via model selection. Second, also different starting values may yield different solutions because the algorithm can converge to a local minimum. The model selection procedure we adopted to find the solutions presented in the following will be discussed in the next section, along with our consideration regarding multiple starting values. Third, a pre-processing step precedes method application. All of the predictor variables are centered and scaled to unit sum of squares. Subsequently, the different predictor blocks are weighted such that the sum of squares are equal across the blocks, in order to account for the differing block sizes.

The estimates retrieved by the method along with the population parameters used to generate the dataset are provided in Table 1. It first shows that the weights $\hat {\mathbf {W}}_{C}$ are found sparse and correctly reflect the population weights zero-nonzero structure. Most of the estimated weights are smaller in magnitude than the population weights because the lasso and group lasso penalties not only enforce sparsity but also shrink the coefficients towards zero. The weights are interpreted as the coefficients in the linear combination that forms the covariates from the predictor variables; $t_{ir}={\sum }_{j} w_{jr}x_{ij}$. Therefore, the weights correctly represent that the first two covariates are distinctive for each of the data blocks while the third is common. The logistic regression coefficients and the intercept $\hat {\mathbf {p}}^{(g)}$ and $\hat {p}_{0}^{(g)}$ are also obtained and are in agreement with the population parameters; the covariate distinctive to the second data block is much less relevant than the other covariates in the classification problem. These coefficients can be combined with the covariates to yield the predicted log-odds; ${\sum }_{r} (\hat {p}^{(g)}_{r}$ $\hat {t}_{ir}) + \hat {p}_{0}^{(g)}= \hat {y}_{i}$. The inverse-logistic function (Eq. 2) is used to transform the $\hat {y}_{i}$ log-odds into predicted probabilities for the categories of the outcome variable; if the probability is larger than 0.5, the class predicted by the model is 1. Let us take an example of the first observation **x**_*C*__1_, the covariate scores of this observation $\hat {\mathbf {t}_{1}} = {\mathbf {x}_{C}}_{1}^{T} \hat {\mathbf {W}_{C}} = [2.875, 0.046, 3.384]^{T}$ are combined with the regression coefficients to get the predicted log odds ${\sum }_{r} (\hat {p}^{(g)}_{r} \hat {t}_{1r}) + \hat {p}_{0}^{(g)} = \log \left (\frac {p(\hat {g_{1}} = 1)}{1 - p(\hat {g_{1}} = 1)} \right ) = 0.862$. Applying the inverse logistic function, the predicted probability for this observation to be classified as 1 is $\frac {1}{1 + e^{-0.862}} = 0.703$. Since this probability is larger than 0.5, we predict the observation as being in class 1, which is indeed true for the first observation in our toy example dataset.

**Table 1:** Population weights, and the solution found by SCD-Cov-logR from the toy example dataset: weights and logistic regression coefficients

**W**_*C*_				$ \hat {\mathbf {W}}_{C} $					
	D1	D2	C		D1	D2	C	Logistic regression coefficients	
Block 1				Block 1				Population	
x1	0.5	0	0	x1	0.358	0	0	D1	− 0.600
x2	0.5	0	0	x2	0.391	0	0	D2	− 0.010
x3	0.5	0	0	x3	0.463	0	0	C	0.800
x4	0.5	0	0	x4	0.475	0	0	intercept	0
x5	0	0	0.354	x5	0	0	0.359		
x6	0	0	0.354	x6	0	0	0.319	Estimated	
x7	0	0	0.354	x7	0	0	0.276	D1	− 0.735
x8	0	0	0.354	x8	0	0	0.233	D2	− 0.072
x9	0	0	0	x9	0	0	0	C	0.907
x10	0	0	0	x10	0	0	0	intercept	− 0.090
x11	0	0	0	x11	0	0	0		
x12	0	0	0	x12	0	0	0		
x13	0	0	0	x13	0	0	0		
x14	0	0	0	x14	0	0	0		
x15	0	0	0	x15	0	0	0		
Block 2				Block 2					
x16	0	0	0.354	x16	0	0	0.358		
x17	0	0	0.354	x17	0	0	0.401		
x18	0	0	0.354	x18	0	0	0.342		
x19	0	0	0.354	x19	0	0	0.307		
x20	0	0.5	0	x20	0	0.483	0		
x21	0	0.5	0	x21	0	0.415	0		
x22	0	0.5	0	x22	0	0.381	0		
x23	0	0.5	0	x23	0	0.453	0		
x24	0	0	0	x24	0	0	0		
x25	0	0	0	x25	0	0	0		
x26	0	0	0	x26	0	0	0		
x27	0	0	0	x27	0	0	0		
x28	0	0	0	x28	0	0	0		
x29	0	0	0	x29	0	0	0		
x30	0	0	0	x30	0	0	0		

The column names D1, D2, and C indicate that the corresponding covariate is defined as being distinctive to block 1, distinctive to block 2 and common

Altogether, examining this solution, it would be concluded that there are two underlying predictor processes that exclusively involve predictor variables of only one of the two data blocks and one process that involves predictors from both data blocks. Predictors x9 to x15 and x24 to x30 are filtered out of the model; they are not related with any of these processes. Only two processes out of the three are important in classifying the binary outcome variable. The predictor process distinctive to the second data block is irrelevant for the classification problem. Concerning the performance of classifying the outcome, the method classified 92 in-sample observations. To gauge the quality of predicting the classes of unseen data, we applied the fitted model to 100 observations of out-of-sample data that were generated from the same population as the in-sample observations. The method was able to classify 92 out-of-sample observations correctly.

### Model selection

The SCD-Cov-logR method involves several (usually) unknown parameters that govern the characteristics of the derived model; the number of covariates *R*, the weighting parameter *α*, the lasso and group lasso parameters *λ*_*L*__*r*_, *λ*_*G*__*r*_ for the sparse weights and the ridge parameter *λ*_*R*_ for the logistic regression coefficients. These parameters are usually tuned in accordance with a certain optimality criterion such as prediction error. Several model selection strategies can be used for different model parameters, while we adopt cross-validation for all of the parameters except for the number of covariates. A straightforward way to administer cross-validation is the grid search that exhaustively compares all possible combinations of the ranges of values for the different parameters in optimizing the criterion of cross-validation error. However, as the current method entails many parameters to be tuned, such a scheme involves a very heavy computational load. Instead, a sequential approach where sets of parameters are tuned in turn can be considered as it was demonstrated to work well for model selection for PCovR (Vervloet, Van Deun, Van den Noortgate, & Ceulemans, 2016) and also for SCD-CovR (Park et al., 2020). In the following, we propose a sequential cross-validation model selection procedure and demonstrate it with the toy example dataset.

The first step of the sequential approach is to determine the number of covariates. This was recommended in a study that compares model selection strategies for PCovR (Vervloet et al., 2016). Park, Ceulemans, and Van Deun (2020) also selected the number of covariates first and obtained models with good performance in SCD-CovR. For finding the number of covariates in SCD-Cov-logR, we first perform PCA on the predictor variables with varying number of principal components. Instead of the well-known scree test that manually looks for an ‘elbow’ in the plot of eigenvalues (representing the amount of variance explained by each principal component) which involves an element of subjectivity, the acceleration factor technique proposed by Raîche, Walls, Magis, Riopel, and Blais (2013) is adopted. It finds the elbow by computing at which point the slope of the graph of eigenvalues change most sharply. The technique retains the principal components that derived prior to the principal component where the sharp change in slopes occurs. The R package “nFactors” is employed for this purpose (Raiche, Magis, & Raiche, 2020).

With the number of covariates fixed, cross-validation is administered to simultaneously select the optimal values of *α* and *λ*_*R*_. For each combination of values, the mean of squared residuals is computed. These residuals are discrepancies between the binary outcome scores of the observations in held-out samples and their corresponding predicted probabilities computed by: $ \frac {1}{n} {{\sum }_{i}^{n}}$ $\left (g_{i} - 1/ \left (1 + e^{-({\mathbf {x}_{C}}_{i}^{T} \hat {\mathbf {W}}_{C} \hat {\mathbf {p}}^{(g)} + \hat {p}_{0}^{(g)})} \right ) \right )^{2} $ where *n* denotes the size of the held-out samples. In the case of the multiclass problem, the residuals are computed by $\frac {1}{n(M-1)} {\sum }^{M-1}_{m}{{\sum }_{i}^{n}}$ $\left [g_{im} - e^{{\mathbf {x}_{C}}_{i}^{T} \hat {\mathbf {W}}_{C} \hat {\mathbf {p}}_{m}^{(g)} + \hat {p_{0}}^{(g)}_{m} } / \left (1 + {\sum }^{M-1}_{m} e^{{{p_{0}}^{(g)}_{m}} + {\mathbf {x}_{C}}_{i}^{T} \mathbf {W}_{C} \mathbf {p}_{m}^{(g)}} \right ) \right ]^{2}$. The one standard error rule (Friedman, Hastie, Tibshirani, & et al. 2001) is adopted, which selects the least complex model within one standard error of the best-performing model. For *α*, higher values are associated with model complexity and overfitting because it places a heavier emphasis on the prediction problem of the outcome which becomes prone to overfitting with increasing number of predictor variables (Babyak, 2004; McNeish, 2015). Similarly, lower values of *λ*_*R*_ are related with overfitting as it leads to high variance of parameter estimates across samples. Therefore, the one standard error rule aims to select the models with the lowest *α* and the highest *λ*_*R*_ values. When the two parameters are not in agreement, the model with lower *α* is preferred over the model with higher *λ*_*R*_ as the former is seen to exert more impact on the final model. Note that the rescaled parameter *β* can be tuned instead of directly tuning for *α*. Higher values of *β* are related to overfitting, in the same manner as for *α*. The one standard error rule would thus choose the models comprised with the lowest *β* and the highest *λ*_*R*_ values in this case.

We tune the sparsity parameters for the weights at the final stage of the model selection procedure because they exert relatively small influences on the fit of the model with respect to both classification or reconstruction of the blocks of predictor variables (de Schipper & Van Deun, 2021; Park et al., 2020). In a paper that examined the efficacy of various model selection strategies for sparsity penalty parameters in sparse PCA that retrieves sparse weights like SCD-Cov-logR, it was reported that even a very sparse model yields good recovery of summary component scores (de Schipper & Van Deun, 2021). The authors advise using cross-validation with the one standard error rule to select the parameters, when the aim of the analysis includes understanding of underlying processes. For our proposed method, the one standard error rule is set up such that the model with the highest values of *λ*_*L*__*r*_ and *λ*_*G*__*r*_ are chosen within models with minimal cross-validation error. Between the two parameters, the model with higher *λ*_*L*__*r*_ is preferred over the model with higher *λ*_*G*__*r*_ because *λ*_*L*__*r*_ encourages the sparse solution in a more direct manner than *λ*_*G*__*r*_. While different values of the parameters can be specified concerning the weights corresponding to each of the *r* th covariate, we usually adopt the same values across multiple covariates to ease the computational burden. Additionally, in choosing the ranges of sparsity parameters to be considered for model selection, values separated by a reasonable interval can be selected between a near-zero value and another value that leads to complete sparsity. One way to choose such an interval is by selecting a sequence of equally spaced values on the log scale, as done in de Schipper and Van Deun (2021) and recommended in Friedman, Hastie, and Tibshirani (2010b).

#### Model selection for the toy example

We demonstrate the model selection procedure by applying it on the toy example dataset. First, PCA is administered to the concatenated set of centered and standardized predictor variables with various numbers of principal components. Figure 4 in Appendix F depicts the variance explained by each component. With the acceleration factor technique, the number of covariates is chosen to be three because the sharpest change in the slopes occurs at the fourth principal component. With the number of covariates fixed, we administered a five-fold cross-validation, simultaneously varying the values of *β* and *λ*_*R*_. Instead of directly controlling the values for *α*, we varied the values for its rescaled version *β*. The parameters *λ*_*L*__*r*_ and *λ*_*G*__*r*_ were fixed at zero for the cross-validation. We considered the values of [0.1, 0.2, 0.3, 0.4, 0.5, 0.6, 0.7, 0.8, 0.9] for *β* and [0.1, 0.5, 1, 3, 5, 10, 30, 50] for *λ*_*R*_. With the one standard error rule, a *β* value of 0.2 and *λ*_*R*_ of 1 was selected. Given these parameters, we finally conducted another five-fold cross-validation for *λ*_*L*__*r*_ and *λ*_*G*__*r*_. The range of [0.5, 1, 5, 7, 10, 15, 30, 45, 100] was employed for *λ*_*L*__*r*_ and [0.1, 0.5, 1, 2, 5, 10] for *λ*_*G*__*r*_. The one standard error rule selected the model with *λ*_*L*__*r*_ = 45 and *λ*_*G*__*r*_ = 2. The solution provided above in Table 1 was obtained by adopting these values for the analysis of the data. It is worth noting that using an exhaustive approach to cross-validation that considers all combinations of these ranges of parameters also resulted in models that are similar to this reported model. The results from this exhaustive approach can be found in Appendix G.

In the above model selection procedures, rational starting values (i.e., the PCovR solution) were used in initializing the SCD-Cov-logR algorithm. To account for the problem of local minima, 20 different sets of random starting values were generated. Using each set of starting values, we conducted the same model selection procedures to find the tuning parameters and the final model estimates. We found that the solution resulted from the rational starting values were associated with the lowest minimum, compared with the other starting values. Comparing the estimates obtained by different starting values, although some starting values yielded estimates that are quite different from those of the rational starting values, the starting values that resulted in smaller loss led to estimates that are very similar to those of the rational starting values. These estimates also correctly classified the same numbers of in-sample and out-of-sample observations as the estimates from the rational starting values. Since the rational starting values led to the lowest minimum, we reported these estimates in the previous section. It also seems sensible that the rational starting values from PCovR finds a lower minimum because the data was generated from a clear PCovR model structure (as seen in the Simulation Study section). However, in practice, it is recommended to adopt multiple random starting values and the rational values to initialize the algorithm and subsequently choose the solution that attains the lowest minimum. This applies especially if the underlying true model structure is unknown, unlike for the current toy example.

### Related methods

SCD-Cov-logR is a classification method with three main objectives. It (a) classifies a categorical outcome, (b) recovers the underlying common and distinctive predictor processes via dimension reduction, and (c) derives sparse weights and therefore interpretable covariates. The method offers a solution that achieves all of these objectives in a flexible manner such that the user can emphasize one goal over another according to the research aim. In this section, we will present two methods that are related to SCD-Cov-logR, in the sense that they target a similar set of goals. Alongside, regularized logistic regression is also discussed as a benchmark method for classification with a large set of predictors.

#### PCR (logistic regression)

A commonly used method that aims both at classification and modeling the variation in the block of predictors is based on principal component regression (PCR; see Jolliffe, 1982). This method first performs PCA on the predictors and then, in a second and separate step, builds a classification model using the retrieved components as the predictor variables. In order to derive common and distinctive processes from multiblock data, the PCA step can be conducted with SCaDS (de Schipper & Van Deun, 2018). We will refer to this two-step approach of SCaDS followed by logistic regression by SCaDS-logR. As discussed above, this is the special case of SCD-Cov-logR with the weighting parameter *α* is specified at zero. Hence, it addresses the same research goals of SCD-Cov-logR, except that it does not take the outcome variable into consideration when deriving the components. Due to this, the underlying processes that play important roles for the outcome variable rather than the predictor variables may be omitted (Vervloet et al., 2016).

#### DIABLO

Data Integration Analysis for Biomarker discovery using a Latent component method for Omics (DIABLO; Singh et al.,, 2016) is a partial least squares (PLS)-based framework that addresses the multiple aims of prediction and sparse modeling of the variation in the predictors. PLS (Wold, 1982; Wold, Martens, & Wold, 1983) is a widely used method that has the same model structures as PCovR; it finds components that represent the underlying processes among the predictors while predicting the outcome variable. PLS can also be seen as an approach to structural equation modeling (SEM) when complex models are built without being mainly guided by theory (Tenenhaus, Tenenhaus, & Groenen, 2017). DIABLO is an extension of PLS that jointly analyzes multiple predictor blocks and obtains sparse components. Simultaneously, these sparse components explain the variation in the outcome variable. Therefore, DIABLO meets all of the research aims of SCD-Cov-logR. While our proposed method treats the multiblock problem by concatenating the predictor matrix to construct a single model that covers several data blocks, DIABLO derives one model separately for each data block; predictions from each model are accumulated via majority voting to give the overall classification. Therefore, DIABLO can be seen to only find components that are distinctive to each block. However, it is possible to specify how correlated these components built on each block would be. This would encourage capturing of the variance accounted for by common predictor processes, although they may not be explicitly obtained. Singh et al., (2016) demonstrated that when building a classification model for breast cancer subtypes with predictors from multiple data blocks (mRNA, miRNA, methylation and proteins) from The Cancer Genome Atlas (TCGA), DIABLO was able to select more variables that are strongly correlated with each other than elastic net regression.

Another core difference between SCD-Cov-logR and DIABLO lies with the parameter *α* that balances between reconstruction of the predictors and prediction of the outcome variable. PLS-based methods do not offer such an option and tend to lean closer to a PCovR model emphasizing prediction, this is *α* close to one (Vervloet et al., 2016; Van Deun et al., 2018). Furthermore, methods based on PLS are often more prone to overfitting than those derived from PCovR, which in turn results in a diminished quality of out-of-sample prediction. The results from Park et al., (2020) demonstrated this pattern of results in a multiblock regression setting.

Moreover, DIABLO does not adopt a generalized linear model framework to treat the classification of categorical outcome variables. Instead, when constructing a classification model, DIABLO adopts a simple heuristic where the categorical outcome is coded into a binary matrix with each column indicating the membership of the observation unit in a certain class. The classification model is then estimated in the same manner as the regression model by treating the binary matrix as continuous outcome variables. Among the fitted values given for each of the classes, the class that corresponds to the largest fitted value is the class determined by the DIABLO model. This approach of administering PLS for a classification problem has also been shown to be equivalent to performing discriminant analysis (Barker and Rayens, 2003). There are PLS methods that are formulated in combination with the generalized linear model framework such that a logistic regression model can be constructed (Ding & Gentleman, 2005; Chung & Keles, 2010), but these methods are only suitable for the analysis of a single data block. Additionally, Lê Cao, Boitard, and Besse (2011) reported that this approach performs comparatively with the binary indicator matrix approach of DIABLO.

#### Regularized logistic regression

Regularized logistic regression is a logistic regression method that performs variable selection (Friedman et al., 2010b). Due to the regularization penalties, the method can also be applied to high dimensional datasets. Hence, it can be considered as a benchmark method for classification in the setting of many predictors, being actively applied in behavioral sciences; for example to detect psychological symptom patterns from large-scale questionnaires (Tutun et al., 2019) and to classify different emotions using EEG signal patterns (Chen et al., 2020). However, since it does not extract covariates or factors, the method does not meet all of the aims of SCD-Cov-logR such as identifying the underlying processes governing the predictors.

#### Toy example illustration

In order to compare the two related methods that share the goals of SCD-Cov-logR, we administered them along with the benchmark of regularized logistic regression on the toy example dataset. As the population model parameters are known, we configured the methods such that they return the solutions that reflect the population model structure as closely as possible. For regularized logistic regression, the lasso penalty parameter was tuned by cross-validation, as it is not possible for the method to derive the covariate structures. For principal component (logistic) regression, we administered SCaDS (de Schipper & Van Deun, 2018) on the predictor matrix with three components. Lasso and group lasso parameters were chosen such that they reflect the population model. The outcome variable was regressed on the derived sparse principal components via logistic regression.

In order to fit the DIABLO model in accordance with the population model such that the common and distinctive predictor processes can be explicitly found, we fitted a one-component model separately from each of the two data blocks which would match the two distinctive covariates generated. For the common covariate, we constructed a one-component model from a supermatrix that concatenates the two data blocks. These components across the blocks were specified to be uncorrelated, as the true covariates were defined to be uncorrelated. As DIABLO allows the users to specify the number of non-zero weights per component, we specified these in correspondence with the number of non-zero weights in the true weights matrix.

Table 2 presents the estimates resulting from the different methods. The table shows that only the two-step principal component logistic regression approach of SCaDS-logR finds the covariates that perfectly represent the population model structure. DIABLO can find the distinctive covariates, but does not perform well at correctly finding the non-zero parameters. It is difficult to interpret the regularized logistic regression coefficients as they do not go hand-in-hand with the population model. However, it can be seen that the predictors that do not have any relations with the covariates were filtered out, yet, also some of the predictors that do have a relation with the covariates were also filtered out.

**Table 2:** Estimates provided by PCR, DIABLO and regularized logistic regression

	**W**_*C*_			SCaDS-logR			DIABLO			LogR
	D1	D2	C	D1	D2	C	D1	D2	C	b
Block 1										
x1	0.5	0	0	0.392	0	0	0	0	0	-0.198
x2	0.5	0	0	0.399	0	0	0	0	0	-0.304
x3	0.5	0	0	0.430	0	0	0	0	-013	-0.262
x4	0.5	0	0	0.496	0	0	0	0	0	-0.112
x5	0	0	0.354	0	0	0.328	0.606	0	0.480	0.265
x6	0	0	0.354	0	0	0.291	0.411	0	0.330	0.336
x7	0	0	0.354	0	0	0.262	0.636	0	0.502	0.333
x8	0	0	0.354	0	0	0.217	0.242	0	0.200	0.221
x9	0	0	0	0	0	0	0	0	0	0
x10	0	0	0	0	0	0	0	0	0	0
x11	0	0	0	0	0	0	0	0	0	0
x12	0	0	0	0	0	0	0	0	0	0
x13	0	0	0	0	0	0	0	0	0	0
x14	0	0	0	0	0	0	0	0	0	0
x15	0	0	0	0	0	0	0	0	0	0
Block 2										
x16	0	0	0.354	0	0	0.357	0	0.537	0.364	0.180
x17	0	0	0.354	0	0	0.370	0	0.533	0.353	0.189
x18	0	0	0.354	0	0	0.311	0	0.525	0.335	0.232
x19	0	0	0.354	0	0	0.281	0	0.389	0	0
x20	0	0.5	0	0	0.443	0	0	0	0	0
x21	0	0.5	0	0	0.424	0	0	0	0	0
x22	0	0.5	0	0	0.419	0	0	0	0	0
x23	0	0.5	0	0	0.479	0	0	0	0	0
x24	0	0	0	0	0	0	0	0	0	0
x25	0	0	0	0	0	0	0	0	0	0
x26	0	0	0	0	0	0	0	0	0	0
x27	0	0	0	0	0	0	0	0	0	0
x28	0	0	0	0	0	0	0	0	0	0
x29	0	0	0	0	0	0	0	0	0	0
x30	0	0	0	0	0	0	0	0	0	0

The true weights **W**_*C*_ is also provided as a reference

With respect to the performance to classify the outcome variable, the number of correctly classified in-sample and out-of-sample observations for each of the methods are provided in Table 3. The results pertaining to SCD-Cov-logR are also given to offer comparison. It appears that SCD-Cov-logR and SCaDS-logR lead to comparable and good predictive performances, although the four methods don’t exhibit large differences.

**Table 3:** Number of correctly classified observations provided by PCR, DIABLO and regularized logistic regression

	SCD-Cov-logR	SCaDS-logR	DIABLO	LogR
In-Sample	92	91	83	87
Out-of-Sample	92	92	84	88

Extending this comparative evaluation of the related methods and SCD-Cov-logR to a simulation study requires comparison of the methods on all criteria that reflect the multiple research aims of SCD-Cov-logR. The benchmark regularized logistic regression does not meet this requirement since it fails to meet all of the research aims; it does not uncover underlying predictor processes via structures such as covariates. While both PCR (SCaDS-logR) and DIABLO address the aims, PCR has been compared in previous works against PCovR and showed underperformance in discovering the true covariate structure (Vervloet et al., 2016) and also in prediction of the outcome (Heij, Groenen, & van Dijk, 2007; Tu & Lee, 2019); the reason that PCR falls short is because its components are found without considering the outcome. Moreover, in the setting of multiple predictor blocks, PCovR resulted in better prediction of the outcome when some of the underlying predictor processes important for predicting the outcome only account for a small amount of variance in the predictors (Park et al., 2020). Therefore, in the simulation study section below, we evaluate the performance of our current method against the only competitor that accounts for all criteria, this is DIABLO.

### Toy example multiclass problem

As an additional demonstration for our current method under a multiclass classification problem, we generated a toy example dataset again with a categorical outcome variable with three categories. The characteristics of the data and the underlying model were kept the same as the toy example above, except for the definition of the regression parameters and the number of observation units (*I* = 1000). Appendix H provides further details on the data generating setup. Out of the three categories, the third category was taken as the baseline category in forming the log-odds models. We administered the sequential model selection procedure as done for the binary problem, employing fivefold cross-validation considering the same ranges of parameters as for the binary problem again (see “Model selection”). The following model parameters were selected: *R* = 3,*β* = 0.1,*λ*_*R*_ = 0.1,*λ*_*L*__*r*_ = 100 and *λ*_*G*__*r*_ = 10. Table 4 shows the solution together with the defined population parameters used to generate the data. It can be seen that the estimated weights correctly represent the true underlying weights. The logistic regression coefficients found are also in agreement with the population parameters; two covariates important for discerning the categories from the third (baseline) category are correctly picked out. Moreover, the constructed model classified 842 in-sample observations and 845 out-of-sample observations correctly (both out of 1000 total observations).

**Table 4:** Population parameters and the solution found by SCD-Cov-logR from the toy example multiclass dataset

**W**_*C*_				$ \hat {\mathbf {W}}_{C} $				Logistic regression coefficients		
	D1	D2	C	D1	D2	C		1	2	
Block 1				Block 1				Population		
x1	0.5	0	0	x1	0.485	0	0	D1	0.600	0.950
x2	0.5	0	0	x2	0.485	0	0	D2	0.010	0.312
x3	0.5	0	0	x3	0.475	0	0	C	-0.800	0.010
x4	0.5	0	0	x4	0.476	0	0	intercept	0	0
x5	0	0	0.354	x5	0	0	0.345			
x6	0	0	0.354	x6	0	0	0.344	Estimated		
x7	0	0	0.354	x7	0	0	0.348	D1	1.843	2.865
x8	0	0	0.354	x8	0	0	0.338	D2	-0.026	0.941
x9	0	0	0	x9	0	0	0	C	-1.966	0.015
x10	0	0	0	x10	0	0	0	intercept	0.033	-0.025
x11	0	0	0	x11	0	0	0			
x12	0	0	0	x12	0	0	0			
x13	0	0	0	x13	0	0	0			
x14	0	0	0	x14	0	0	0			
x15	0	0	0	x15	0	0	0			
Block 2				Block 2						
x16	0	0	0.354	x16	0	0	0.350			
x17	0	0	0.354	x17	0	0	0.345			
x18	0	0	0.354	x18	0	0	0.348			
x19	0	0	0.354	x19	0	0	0.349			
x20	0	0.5	0	x20	0	0.482	0			
x21	0	0.5	0	x21	0	0.475	0			
x22	0	0.5	0	x22	0	0.480	0			
x23	0	0.5	0	x23	0	0.482	0			
x24	0	0	0	x24	0	0	0			
x25	0	0	0	x25	0	0	0			
x26	0	0	0	x26	0	0	0			
x27	0	0	0	x27	0	0	0			
x28	0	0	0	x28	0	0	0			
x29	0	0	0	x29	0	0	0			
x30	0	0	0	x30	0	0	0			

The column names D1, D2 and C indicate that the corresponding covariate is defined as being distinctive to block 1, distinctive to block 2 and common. The third category is chosen as the baseline category; the regression coefficients construct the log-odds of the first or the second category as opposed to the third

## Simulation study

Through a simulation study, we study the performance of the SCD-Cov-logR and DIABLO with respect to retrieval of the underlying processes and the classification of a binary outcome variable. We focus on the binary classification problem as the multiclass problem is a direct extension of the binary problem; it is expected that the insights obtained from the binary problem to be applicable for the multiclass problem. We hypothesize that SCD-Cov-logR would be better at out-of-sample classification than DIABLO as it is less susceptible to overfitting. SCD-Cov-logR would also provide models that better reflect the true underlying predictor processes as it allows a good balance between explaining the predictors and the outcome via the weighting parameter.

### Design and procedure

We relied on the data generating setup presented by Chung and Keles (2010) which was used for examining the performance of several variants of sparse PLS that were set up to address the classification problem. Fixing the number of observations *I* to 100, the setup was modified such that two blocks of predictor variables were generated from three underlying covariates. One distinctive covariate per each predictor block was defined, while the remaining covariate reflected a common process involving both of the blocks. The three covariates were defined to differ in relevance for predicting the outcome variable, in that only two of them were defined as being relevant. We generated *J* = 200 predictor variables (100 per data block) for the high dimensional setting and *J* = 30 (15 per data block) for the low dimensional. The following setup was used:

11 $$ \begin{array}{@{}rcl@{}} && \mathbf{T} \sim \mathcal{M}\mathcal{V}\mathcal{N}(\mathbf{0}, \mathbf{\Sigma} = 50^{2} \mathbf{I}_{3}) \\ && \mathbf{E} \sim \mathcal{M}\mathcal{V}\mathcal{N}(\mathbf{0}, \mathbf{\Sigma}_{E} = \sigma^{2} \mathbf{I}_{J}) \\ && \mathbf{X}{_{C}} \gets \mathbf{T} \mathbf{W}_{C}^{T} + \mathbf{E} \\ && \mathbf{z} \gets 1 / (1 + exp(-\mathbf{T} \mathbf{p}^{(g)})) \\ && g_{i} \sim Bernoulli(z_{i}) \end{array} $$

**T** is a *I* × 3 covariate scores matrix drawn from a multivariate normal distribution defined with the mean vector fixed to **0** and a diagonal covariance matrix Σ with all of its diagonal elements fixed at 50^2^. The three covariates are therefore uncorrelated. The columns of the *J* × 3 weights matrix **W**_*C*_ is defined such that they reflect the defined common or distinctive nature of the corresponding covariates. For example, weights corresponding to a covariate distinctive to the first predictor block, are non-zero only for predictors in the first block while the remaining weights corresponding to predictors in the second block are all zero. Likewise, for a common covariate, non-zero weights are defined for predictors in both blocks. On top of these zero weights that determine the common or distinctive nature of the covariates, further sparsity is added by defining more elements of **W**_*C*_ as zeros. The sparsity levels of the weights matrix is fixed at 82% and 85% for low and high dimensional settings, respectively. It is important to note that the weights matrix was constructed such that it is column-orthogonal: $\mathbf {W}_{C}^{T} \mathbf {W}_{C} = \mathbf {I}_{R}$. Together with the covariates **T** which are orthogonally defined, this model corresponds to the well-known PCA decomposition where the weights are equal to the loadings (Guerra-Urzola et al., 2021, for discussion;). This is why the weights $\mathbf {W}_{C}^{T}$ in Eq. 11 linearly combine the covariates **T** to generate the predictors **X**_*C*_ in the same manner as loadings in PCA decomposition. An example of the population weights matrix in a low dimensional setting is presented in “Toy example” (Table 1) along with the toy example dataset, and the weights are defined in a similar manner for a high dimensional setting.

The predictors **X**_*C*_ are generated by multiplying the covariate scores matrix with the weights matrix and adding random error on top. The residual matrix **E** is generated from a multivariate normal distribution with zero mean vector and a diagonal covariance matrix Σ_*E*_ such that the residuals are uncorrelated with each other and also with the covariate scores. The variance of the error variables is adjusted according to one of the manipulated design factors of the simulation study: proportion of variance in **X**_*C*_ explained by the underlying covariates. **p**^(*g*)^ indicates the regression coefficients. *g*_*i*_ is sampled from a Bernoulli distribution with the probability defined by the linear combination of **T** and **p**^(*g*)^ transformed by the inverse-logitic function (see Eq. 2).

Based on this data generating model, we manipulated three data characteristics which are listed in the overview below. The different levels taken by these manipulated factors are provided between square brackets.

#### Study setup

1. Number of predictors *J*_*k*_ in each block: [100], [15]
2. Covariates relevant to the response **g**: [D1, D2], [D1, C]
3. Proportion of variance in **X**_*C*_ explained by the covariates: [0.8], [0.5], [0.2]

The number of predictors manipulated by the first design factor determines whether the dataset would be low or high-dimensional. The second design factor indicates which covariates are relevant for the classification of the binary outcome with D1, D2, and C denoting the two distinctive and the common covariate, respectively. The relevance of the covariates is manipulated by specification of regression coefficients **p**^(*g*)^, which equals [0.60,− 0.80,0.01] and [0.60,0.01,− 0.80] for the two levels respectively. For the first level, the two distinctive covariates are made relevant in explaining the outcome variable, while the covariate distinctive to the first block and the common covariate are relevant in the second level. As stated above, the proportion of variance in the predictors accounted for by the covariates is controlled by the variance of the error variables **E**. Fully crossing these factors and generating 50 datasets per condition, 2 × 2 × 3 × 50 = 600 datasets were produced.

Two different analyses were administered to each of these datasets: SCD-Cov-logR and DIABLO. As done for DIABO for the toy example dataset, a one-component model was fitted for each of the two data blocks to match the two distinctive covariates generated. For the common covariate, we constructed a one-component model from a supermatrix that concatenates the two data blocks.

### Model selection

As the true underlying structure of the datasets is already known, several tuning parameters were tailored to correspond to the true structure. For SCD-Cov-logR, the number of covariates was fixed at three. The weighting parameter *α* and the ridge penalty parameter *λ*_*R*_ that regularizes the logistic regression coefficients were tuned together via fivefold cross-validation. As done in the toy example in “Model selection”, we used the rescaled weighting parameter *β* instead of *α*. The ranges of [0.3, 0.4, 0.5, 0.6, 0.7, 0.8, 0.9] and [0.5, 1, 5, 10, 30, 50] respectively were used for *β* and *λ*_*R*_. We adopted the 1 standard error (SE) rule to select a set of parameters which provides the most general model among the set of parameters yielding errors within 1 SE from minimum cross-validation error. We chose the lowest *β* and the highest *λ*_*R*_. For the toy example, the lasso *λ*_*L*__*r*_ and the group lasso *λ*_*G*__*r*_ parameters were fixed at zero while tuning *β* and *λ*_*R*_. Instead, for the simulation study, they were fixed differently for various conditions of the simulation study to encourage retrieval of one common and two distinctive covariates (Appendix I).

Finally, with values of *β* and *λ*_*R*_ fixed, in order to find the parameters *λ*_*L*__*r*_ and *λ*_*G*__*r*_ that match the population weights structure the closest, we fitted the method with a range of values for *λ*_*L*__*r*_ and *λ*_*G*__*r*_. The ranges of [3, 5, 10, 15, 20, 30, 50, 80] and [0.5, 1, 2, 3, 5, 10] were adopted respectively for *λ*_*L*__*r*_ and *λ*_*G*__*r*_. As in the toy example dataset, the datasets have been generated such that a PCovR model underlies the true sparse model structure. This means that the rational starting values are likely to provide a more optimal solution than random starting values. Therefore, we only employed the rational starting values based on PCovR.

For DIABLO, we specified the number of nonzero weights according to the defined model structure. As done for the toy example dataset, the components from different blocks were fitted such that they are not correlated. This is sensible because the true covariates are generated to be uncorrelated from each other.

### Evaluation criteria

Because the methods have several objectives, including recovery of the underlying processes and classification of a binary outcome, two measures are used to study performance of the methods in relation to each of these objectives. The performance measures are:

1. Out-of-sample balanced error rate (BER): (false positive rate + false negative rate)/2.
2. Correct weights classification rate: proportion of the weights correctly classified as zero and non-zero elements relative to the total number of coefficients.

An independent test set (of 100 observation units) needed for computing the out-of-sample BER was generated following the same data generating procedures as the data used for model-fitting. A BER equal to zero indicates a perfect classification. The correct weights classification rate represents the method’s ability in retrieving the underlying processes. SCD-Cov-logR provides weights matrix $\hat {\mathbf {W}}_{C}$ of size ${\sum }^{2}_{k = 1} J_k \times R$ which covers the entire set of the multiblock predictors. For the weights provided by SCD-Cov-logR, we first computed Tucker congruence (Tucker, 1951) between the columns of the true **W**_*C*_ matrix and those of the estimated $\hat {\mathbf {W}}_{C}$ matrix. After matching the columns that resulted in the highest Tucker congruence to account for the permutational freedom of the covariates, the correct classification rate was calculated from the matching pairs of true and estimated **W**_*C*_ columns.

On the other hand, for DIABLO, one component each was estimated for the two predictor blocks and the concatenated supermatrix. Components derived from the individual predictor blocks naturally correspond to the true distinctive covariates. In order to calculate the correct classification rate, the weights estimated for these estimated components were compared against true weights that correspond to the true distinctive covariates. Likewise, the weights found from the concatenated supermatrix were compared against the true weights corresponding to the common covariate.

### Results

#### Out-of-sample BER

**Fig. 1:**
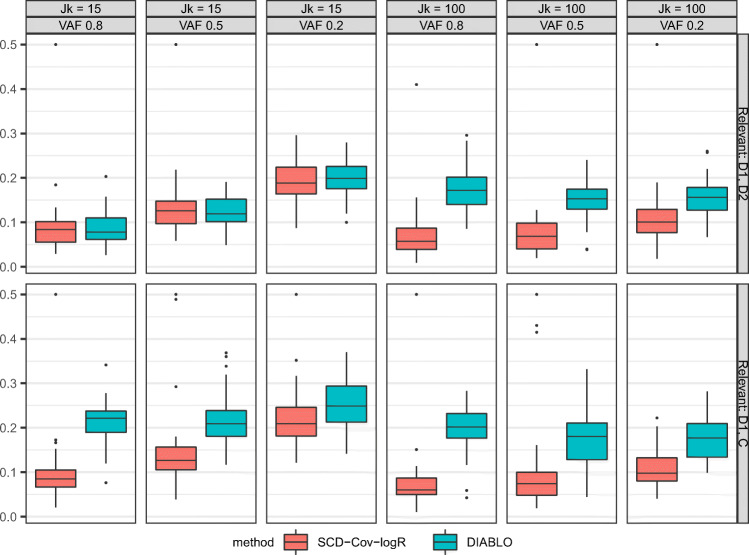
Box plots of the out-of-sample BER; each panel corresponds to one of the 12 conditions. The *column panels* indicate the number of predictors in each data block and the proportion of variance accounted for by the underlying processes. The *row panels* indicate the two covariates relevant for the outcome variable; “D1”, “D2” and “C” refer to the covariate distinctive to the first block, the covariate distinctive to the second block and the common covariate, respectively

We first examine the performance of the two methods concerning the prediction for new data. The estimates obtained by the methods from the training dataset are applied on the out-of-sample test set generated under equal conditions. The results from our simulation study arranged for each condition are displayed in Fig. 1. It can first be seen that SCD-Cov-logR resulted in the smaller out-of-sample BER in almost all of the conditions. With regards to the manipulated design factors, the relevance of the covariates seems to have played an important role in different performances among the methods. When the two distinctive covariates are defined as being relevant, the discrepancy in the methods is smaller, but with the covariate distinctive to the first block and the common covariate relevant, the outperformance of SCD-Cov-logR stands out more prominently. The proportion of variance in **X**_*C*_ accounted for by the covariates resulted in the ‘main effect’ - with smaller proportion leading to higher BER for all of the methods. Finally, it appears that the discrepancy in the performance of the methods is larger when the dataset is high-dimensional. Overall, we conclude that SCD-Cov-logR outperforms DIABLO at predicting the classes of new observations. However, the methods present more comparable performance when the processes relevant for classification are distinctive, under low dimensionality.

#### Correct weights classification rate

**Fig. 2:**
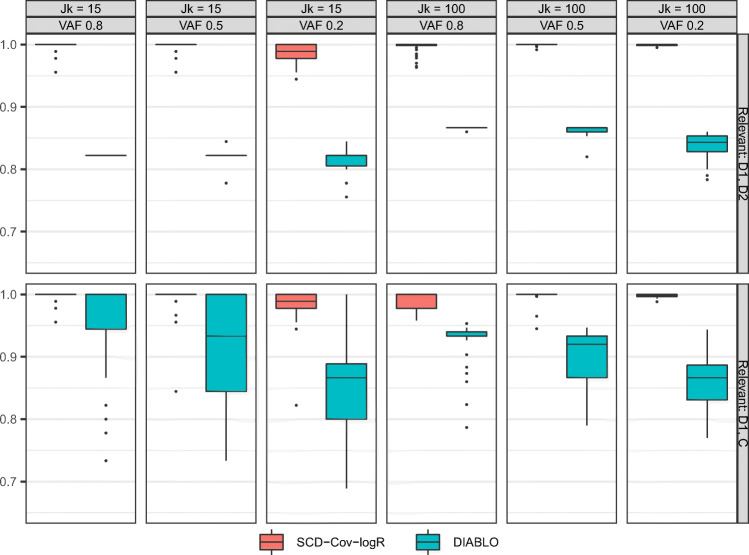
Box plots of the correct weight classification rate; each panel corresponds to one of the 12 conditions. The column panels indicate the number of predictors in each data block and the proportion of variance accounted for by the underlying processes. The row columns refer to the two covariates relevant for the outcome variable; “D1”, “D2” and “C” refer to the covariate distinctive to the first block, the covariate distinctive to the second block and the common covariate, respectively

Figure 2 presents the outcome of the correct weights classification rate. Across all of the conditions, SCD-Cov-logR resulted in the higher of correct classification. It is also noteworthy that the classification rate for the method is mostly above 0.95. The figure shows the influence of the relevance of the underlying covariates and its interaction with the other manipulated data circumstances. When the two distinctive covariates were relevant, regardless of the dimensionality, SCD-Cov-logR resulted in a much higher classification rate than DIABLO. On the other hand, when the covariate distinctive to the second data block was defined irrelevant, DIABLO’s performance was closer to SCD-Cov-logR’s in the conditions with more variance of the predictors explained and with 15 predictor variables per block. In conclusion, SCD-Cov-logR is better than DIABLO at correctly retrieving the underlying population weights.

## Illustration: 500 Family data

### Dataset and pre-processing

We demonstrate an example use of SCD-Cov-logR by administering the method on an empirical dataset. We adopted the dataset from the 500 Family Study (Schneider & Waite, 2008) which investigated into how work impacts the well-being of parents and children in American middle-class families. Questionnaire data from different members of the same family were collected. We computed sum scores from questionnaire items that refer to the same construct. These scores concern the feelings of the family members, their recent mutual activities and how they perceive their relationship. 24 sum score variables were computed and are used as predictors in constructing the SCD-Cov-logR model. They can be found in Table 5. Eight of the predictors pertain to responses from the mother, another eight to responses from the father and lastly six predictors are based on the responses of the child. The dataset therefore is comprised of three blocks according to the member of the family, and each observation unit refers to a family. All of the predictors were centered and standardized. Since the blocks have different sizes, they were weighted such that the sum of squares is equal across blocks.

**Table 5:** Weights and logistic regression coefficients derived by SCD-Cov-logR from the 500 family dataset

$ \hat {\mathbf {W}}_{C} $				
	Child	Parents	Logistic regression coefficients	
Mother			Estimated	
Relationship with partners	0	0.276	Child	0.288
Argue with partners	0	0.269	Parents	0.034
Child’s bright future	0	0	Intercept	-0.007
Activities with children	0	0		
Feeling about parenting	0	0.188		
Comunication with children	0	0.357		
Argue with children	0	0.171		
Confidence about oneself	0	0.406		
Father				
Relationship with partners	0	0.091		
Argue with partners	0	0.183		
Child’s bright future	0	0		
Activities with children	0	0		
Feeling about parenting	0	0		
Comunication with children	0	0		
Argue with children	0	0.210		
Confidence about oneself	0	0.050		
Child				
Self-confidence/esteem	0.285	0		
Social life and extracurricular	0.336	0		
Importance of friendship	0.459	0		
Self Image	0.381	0		
Happiness	0.374	0		
Confidence about the future	0.281	0		

The covariate labels heading the columns of the table with weights and the rows of the table with logistic regression coefficients indicate which data blocks the corresponding covariate is associated with

The families are categorized into two groups according to the child’s most recent grade at school. The family with the child with a grade B or higher is classified as having academic overachievement (coded as 1), while grade C or lower is classified as underachievement (coded as 0). We excluded the families with missing values on any of the predictor variables, and made a random subset selection of 58 families in order to obtain a balance between the size of two categories. We conducted SCD-Cov-logR to target this classification problem of academic underachievement while simultaneously constructing a model that describes the underlying common and distinctive processes of the three predictor blocks.

### Model selection

We employed the sequential cross-validation model selection strategy discussed in “Model selection” applied to the toy example dataset. Moreover, 50 sets of random starting values were employed alongside the rational starting values in conducting the model selection and final model fitting.

First, the number of covariates was found by administering PCA on the predictor matrix. By using the acceleration factor technique, we found that when going from 1 to 2 principal components, the amount of variance explained by the principal components changes the most drastically (Figure in the Appendix J). With the number of covariates determined at two, we carry out the cross-validation to select the other tuning parameters. The different sets of starting values were introduced at this stage. The complete process of model selection and model fitting was conducted for each set of starting values. The resulting solutions from 50 random starting values and 1 rational starting value were compared in terms of the value of the loss function: The solution with the smallest loss was retained as the final solution.

The cross-validation procedures administered for each of the starting values were as the following: first, 20-fold cross-validation was conducted with varying values of the rescaled weighting parameter *β* and *λ*_*R*_. At this stage, the tuning parameters *λ*_*L*__*r*_ and *λ*_*G*__*r*_ were fixed at zero for the cross-validation. We considered the values of [0.1, 0.2, 0.3, 0.4, 0.5, 0.6, 0.7, 0.8, 0.9] for *β* and [0.01, 0.05, 0.1, 0.5, 1, 2, 5, 10, 15, 20] for *λ*_*R*_. Using the one standard error rule, values of *β* and *λ*_*R*_ are selected. Given these selected values, the second sequence of 20-fold cross-validation for *λ*_*L*__*r*_ and *λ*_*G*__*r*_ was conducted. With the ranges of [0, 0.05, 0.1, 0.3, 0.5, 1, 3, 5, 7, 10, 15, 20, 30, 50] adopted for both parameters, the same parameter value was used concerning the two covariates. We used the one standard error rule again to choose the values of *λ*_*L*__*r*_ and *λ*_*G*__*r*_, completing the model selection procedure.

Similar to the toy example dataset, a smaller minimum was achieved by the set of rational starting values. The final values for the tuning parameters selected through the sequential procedure were: *β* = 0.1, *λ*_*R*_ = 2, *λ*_*L*__*r*_ = 10, *λ*_*G*__*r*_ = 10. The final model estimates obtained are presented in Table 5.

### Results

The estimated weights matrix from Table 5 show that there are two predictive processes for the child’s academic achievement. The first component is distinctive to the child block and is associated with all of the variables from the data block. It appears that all of the variables in the child block have an impact in the academic achievement. On the other hand, the second component is locally common, involving several variables from the mother and the father blocks but not from the child block. Observing the weights from the second covariate, it can be seen that parents’ high confidence in the child’s future and the amount of activities they partake with the child are not important in predicting the child’s academic achievement. Also, according to this model, the father’s positive feeling about parenting and his level of comunication do not exert strong influence in the child’s academic achievement. Moreover, the logistic regression coefficients suggest that the Child covariate is much more relevant in predicting child’s academic achievement group. It appears that the attitudes that the children themselves have are the most important in leading to academic overachievement.

**Fig. 3:**
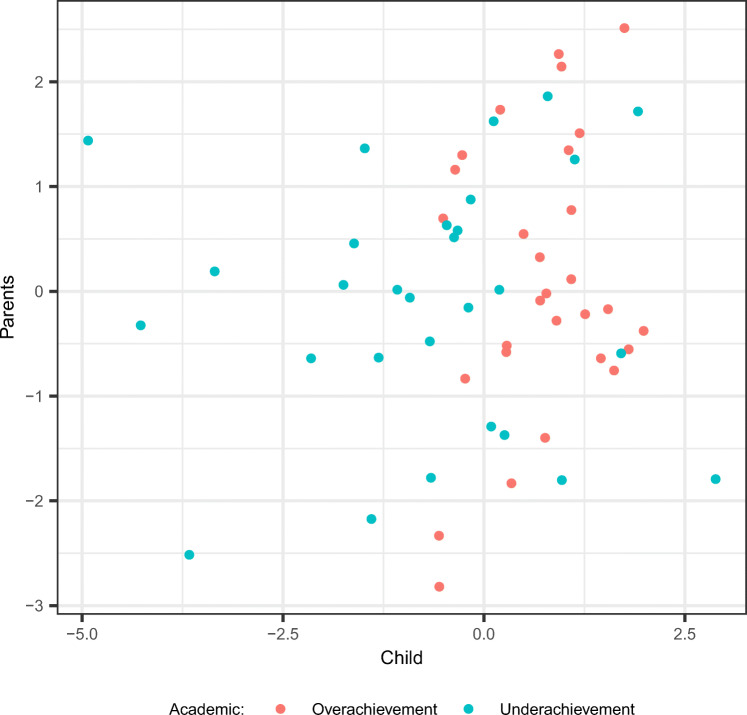
Scatterplot of the two covariates found by SCD-Cov-logR. The colors represent the academic achievement of the child

**Fig. 4:**
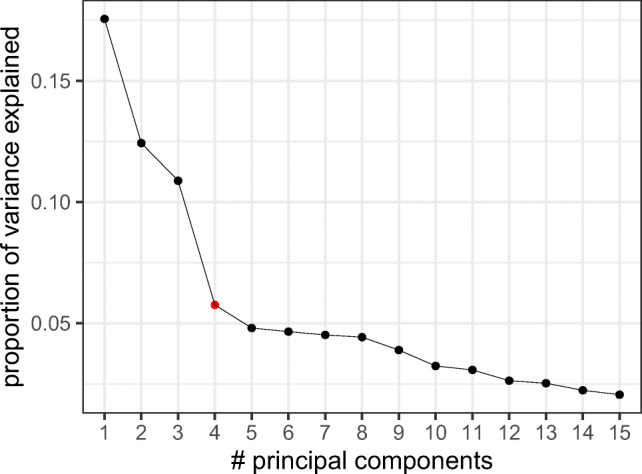
It can be seen that the sharpest change of slopes occurs at four principal components. Three components are therefore retained in the model

The covariate scores of the 58 families can be seen in Fig. 3 which presents a fair separation of the two categories of the families. With the observations separated along the X-axis, It can be seen that the Child covariate plays a more important role in separating the two groups. This is in line with the small magnitude of the coefficient corresponding to the Parents covariate. Out of the 58 families, the final model classifies 43 families correctly. In order to also examine the classification performance of the model on out-of-sample data, we performed a leave-one-out cross-validation which resulted in 40 families being correctly classified. Together, this implies that the model showed about 70% of classification accuracy for both in-sample and out-of-sample observations.

To obtain more comparative insight about the quality of the method under this empirical dataset, we administered the related methods discussed in the methods section; regularized logistic regression, PCR (SCaDS-logR) and DIABLO. The PCA step for the PCR was conducted with SCaDS to tackle the multiblock nature of the data, as demonstrated with the toy example dataset in “Related methods” The number of components for SCaDS was set at two, so that the model is comparable to the SCD-Cov-logR model constructed with two covariates. The lasso and group lasso parameters governing the sparseness of SCaDS weights were selected with 20-fold cross-validation with the one standard error rule. Similarly, a two-component model was estimated with DIABLO. The number of non-zero weights to be estimated per component was tuned via 20-fold cross-validation. Lastly, the lasso parameter for regularized logistic regression was also chosen with 20-fold cross-validation. Table 6 provides the number of correctly classified in-sample observations from each of the methods. As done for SCD-Cov-logR, leave-one-out cross-validation was conducted to gauge the out-of-sample classification quality. These results are also provided in the table. It can be seen that the four methods led to very comparable performances with respect to prediction. The estimates derived by the methods are provided in Appendix K and they can be inspected to understand the constructed models. It was found that only SCaDS-logR identified predictive processes concerted by several predictors, akin to the covariates of SCD-Cov-logR. Both regularized logistic regression and DIABLO found a very sparse model with only two non-zero coefficients.

**Table 6:** Number of correctly classified observations (out of the total 58) provided by SCD-Cov-logR, PCR, DIABLO and regularized logistic regression

	SCD-Cov-logR	SCaDS-logR	DIABLO	LogR
In-sample	43	43	44	43
Out-of-sample (leave-one-out CV)	40	41	38	40

The out-of-sample classification is computed via leave-out-out cross-validation

In conclusion, our proposed method is capable in meeting its goals when applied to an empirical dataset. The method identifies common and distinctive covariates and weights that are interpretable. At the same time, the method is able to correctly classify both the samples used for fitting the model and new samples.

## Discussion

A multitude of goals are of interest when building a classification model from a multiblock dataset. The common and distinctive predictor processes need to be identified in an interpretable manner while classifying the outcome variable. We have proposed the method of SCD-Cov-logR that fulfills these goals in a simultaneous manner. We have evaluated the method comparatively against DIABLO; a multiblock variant of PLS. It was found that the proposed method outperforms DIABLO in the objectives that the methods attain: quality of classification and retrieval of weights that are used to understand the underlying processes. Moreover, while DIABLO requires prior information for identifying the common and distinctive processes, our proposed method is able to explore these structures without explicit specification.

In particular, SCD-Cov-logR was found to be considerably better than DIABLO in accurately retrieving the weights matrix. This finding is in line with existing literature that compares between the methodologies of PLS and PCovR. Methods based on PLS tend to place heavier focus on prediction of the outcome variables, as opposed to exploring the structure of the underlying predictor processes. In contrast, the weighting parameter *α* in the PCovR methods helps to attain a good balance between emphasizing the predictor or the outcome variables. In the current paper, all of the results were based on the rescaled parameter *β* tuned via cross-validation. This suggests that the parameter can be used effectively in a purely data-driven approach.

SCD-Cov-logR also has weaknesses. Model selection is an inherent challenge since the method requires many parameters to be tuned to meet its multiple research aims. There are in total 5 parameters to be selected and they all play an important role in shaping the retrieved model. Adopting the solution recommended by Vervloet, Van Deun, Van den Noortgate, and Ceulemans (2016), the current paper suggested a sequential model selection approach where sets of tuning parameters are chosen through cross-validation with the other parameters fixed. Models obtained by this approach led to good results in both simulation experiments and empirical study. We have not visited the model selection problem of our method in great detail as the main purpose of this paper lies within the proposal and illustration of the novel method.

Another remark about the model selection procedure is the optimality criterion used for cross-validation. Throughout the paper, we adopted the sum of squared cross-validation errors concerning the binary outcome variable. This implies that the model selection procedure is conducted only considering the out-of-sample prediction quality. Since our method is not only used for classification of the outcome but also exploring the predictor processes, the optimality criterion for cross-validation can be changed to also include the errors pertaining to the predictor variables. This choice is in the same spirit of the weighting parameter *α*; if the user is interested more in the exploration of the predictor processes, it may be a viable option to look into such an optimality criterion different from what is used in this paper.

In our illustration of the toy data example and the simulation study, DIABLO was fitted in a peculiar manner to allow for derivation of the distinctive and common covariates. However, in practice, there may be other ways of specifying the method. For example, a supermatrix of concatenated blocks can be provided as the only input dataset and a single DIABLO model can be constructed on it.^3^ We have explored into such a specification, and found that it results in consistent underperformance compared to SCD-Cov-logR with respect to prediction and retrieval of population parameters. It also has a tendency to only find common covariates.

Finally, the method and the current paper suggest several future directions of research. It would be a natural extension to broaden the method to encompass generalized linear models. This would allow modeling of outcome variables in diverse nature such as count data. Furthermore, such an extension would allow other related research questions to be addressed. For example, within the high-dimensional multiblock setting, it would be interesting to examine the impact of using a generalized linear model framework to model the categorical outcome, as opposed to the discriminant analysis approach adopted for DIABLO where the categorical outcome variable is simply changed into a dummy matrix and a linear regression model is fit. Although Lê Cao, Rossouw, Robert-Granié, and Besse (2008) compared the two approaches and reported that they show comparable performance in practice, the comparison has not been conducted in the multiblock data setting. Our proposed method SCD-Cov-logR can also be easily adapted into the linear regression approach using a dummy outcome matrix, if it is found to be useful in certain data circumstances.

## Figures and Tables

**Algorithm 1:**
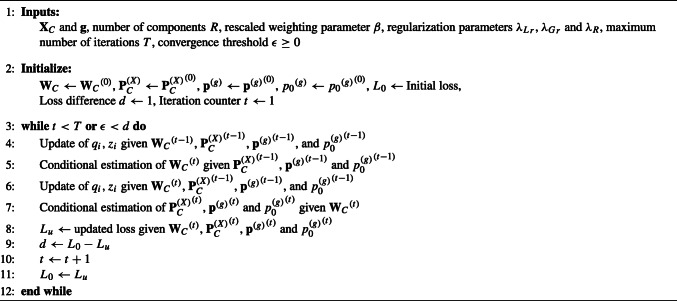
SCD-Cov-logR.

**Algorithm 2:**
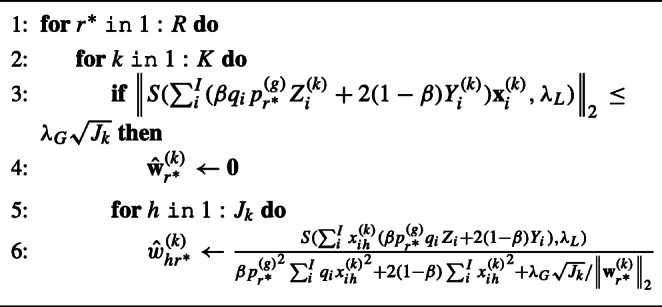
Coordinate descent for sparse group lasso.

**Algorithm 3:**
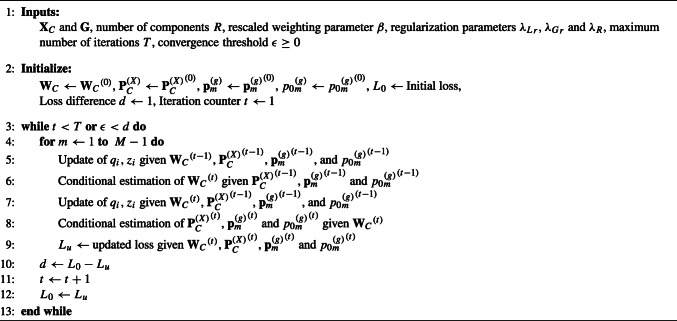
SCD-Cov-logR for multiclass classification.

**Fig. 5:**
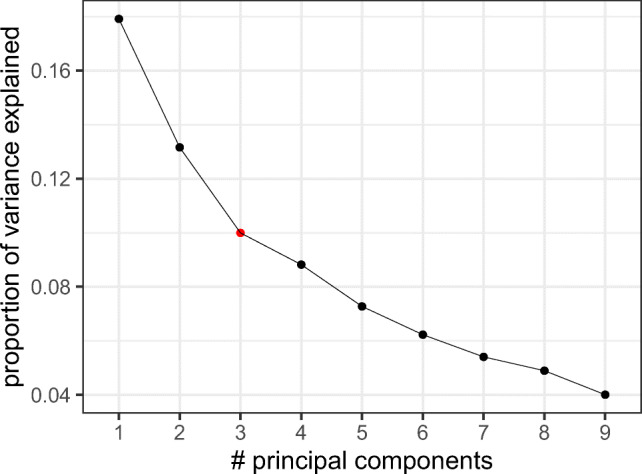
It can be seen that the sharpest change of slopes occurs at three components. Two components are therefore retained in the model

## Data Availability

Code used to generate the results reported in the manuscript is available on GitHub: https://github.com/soogs/SCD-Cov-logR. Publicly available dataset from the 500 Family Study (Schneider & Waite, 2008) was used for empirical illustration.

## Appendix A: SPCovR, SCaDS and SCD-CovR

### A.1: SPCovR

For easier interpretation of the principal covariates and consistency of estimates in the high dimensional settings, regularization penalties have been imposed on the weights from Eq. 5 to lead to sparse PCovR (SPCovR; Van Deun et al.,, 2018). The method finds sparse weights by minimizing the following objective function:

12 $$ \begin{array}{@{}rcl@{}} L(\mathbf{W}_{k}, \mathbf{P}_{k}^{(X)}, \mathbf{p}^{(y)}) & =& \alpha \frac{\left\| \mathbf{y} - \mathbf{X}_{k} \mathbf{W}_{k} \mathbf{p}^{(y)} \right\|_{2}^{2}}{\left\| \mathbf{y} \right\|_{2}^{2}} \\&&+ (1 - \alpha) \frac{\left\| \mathbf{X}_{k} - \mathbf{X}_{k} \mathbf{W}_{k} (\mathbf{P}_{k}^{(X)})^{T} \right\|_{2}^{2}}{\left\| \mathbf{X}_{k} \right\|_{2}^{2}} \\ && + \lambda_{L} \left| \mathbf{W}_{k} \right|_{1} + \lambda_{R} \left\| \mathbf{W}_{k} \right\|_{2}^{2} \end{array} $$

such that $(\mathbf {P}_{k}^{(X)})^{T} \mathbf {P}_{k}^{(X)} = \mathbf {I}_{R}$ and with *λ*_*L*_ ≥ 0, *λ*_*R*_ ≥ 0 and *α* ≥ 0. The regularization parameters are the lasso, with $ \left | \mathbf {W}_{k} \right |_{1} = {\sum }_{j_{k},r} |w_{j_{k}r}|$, and the ridge $ \left \| \mathbf {W}_{k} \right \|_{2}^{2} = {\sum }_{j_{k},r} w_{j_{k}r}^{2}$, together forming the elastic net penalty (Zou & Hastie, 2005). The ridge penalty shrinks the magnitude of the estimates and encourages stable estimation for high-dimensional data, while the lasso penalty is involved in variable selection by shrinking and forcing the estimates to exactly zero. When both penalties are defined at 0, it can be seen that the PCovR formulation (Eq. 5) is retrieved.

### A.2: SCA and SCD-CovR

SPCovR only targets data with a single predictor block and hence do not address the questions associated with multiple predictor blocks. A joint analysis of the *K* predictor blocks can be conducted by imposing a multiblock PCovR model, based on the SCA model (Kiers & Ten Berge, 1989):

13 $$ \begin{array}{@{}rcl@{}} \mathbf{X}_{C} & = & \mathbf{X}_{C} \mathbf{W}_{C} (\mathbf{P}_{C}^{(X)})^{T} + \mathbf{E}^{(X)} \\ \mathbf{y} & = & \mathbf{X}_{C} \mathbf{W}_{C} \mathbf{p}^{(y)} + \mathbf{e}^{(y)} \end{array} $$

where $\mathbf {X}_{C}= \left [ \mathbf {X}_{1}, \ldots , \mathbf {X}_{K} \right ]$ (of size $I \times {\sum }^{K}_{k = 1} J_{k}$) denotes the supermatrix that concatenates the predictor blocks. Consequently, **W**_*C*_ and $\mathbf {P}_{C}^{(X)}$ are weight and loading matrices of size ${\sum }^{K}_{k = 1} J_{k} \times R$. **p**^(*y*)^ indicates a vector of *R* regression coefficients.

When SCA is administered to study the processes underlying the variables without considering the regression problem, the concatenated weights matrix **W**_*C*_ is examined to understand the nature of the components. In order to allow SCA to explicitly distinguish common and distinctive processes and provide a sparse and interpretable solution from high dimensional multiblock datasets, de Schipper and Van Deun (2018) proposed SCaDS. Regularization penalties are imposed upon the weights to force certain elements to zero for handier interpretation, while the **W**_*C*_ matrix is further constrained such that certain components are a priori fixed as being common or distinctive.

Making use of the multiblock PCovR model (Eq. 13) and also combining with SCaDS, SCD-CovR extends SPCovR to allow multiblock analysis. It predicts the outcome, while providing sparse weights that capture the common and distinctive processes in the predictor blocks. SCD-CovR implies minimizing the following objective function:

14 $$ \begin{array}{@{}rcl@{}} L(\mathbf{W}_{C}, \mathbf{P}^{(X)}_{C}, \mathbf{p}^{(y)}) & =& \alpha \frac{\left\| \mathbf{y} - \mathbf{X}_{C} \mathbf{W}_{C} \mathbf{p}^{(y)} \right\|_{2}^{2}}{\left\| \mathbf{y} \right\|_{2}^{2}} \\&&+ (1 - \alpha) \frac{\left\| \mathbf{X}_{C} - \mathbf{X}_{C} \mathbf{W}_{C} (\mathbf{P}^{(X)}_{C})^{T} \right\|_{2}^{2}}{\left\| \mathbf{X}_{C} \right\|_{2}^{2}} \\ && + \lambda_{L} \left| \mathbf{W}_{C} \right|_{1} + \lambda_{R} \left\| \mathbf{W}_{C} \right\|_{2}^{2} \end{array} $$

such that $(\mathbf {P}_{C}^{(X)})^{T} \mathbf {P}_{C}^{(X)} = \mathbf {I}_{R}$, and subject to zero block constraints on **W**_*C*_ that fix weights that correspond to one or several predictor blocks to zero. This implies that the component is determined only by predictors of those blocks for which the weights have not been fixed to zero. Common components are obtained by not placing such zero block constraints on the component. The elastic net penalty and the constraints concerning the weights are the same as imposed in SCaDS. Also, as in SPCovR, the lasso penalty achieves sparseness within the common and distinctive covariates.

## Appendix B: SCD-Cov-logR algorithm

The minimizing solution of Eq. 7 can be found by iteratively reweighted least squares which involves formulating the quadratic approximation of the negative log likelihood given the current estimates of the parameters (Friedman et al., 2010b). The negative log likelihood part of the objective function is as the following:

15 $$ \begin{array}{@{}rcl@{}} L_{logr}(\mathbf{W}_{C}, \mathbf{p}^{(g)}, p^{(g)}_{0}) \!&=&\! -{\sum\limits_{i}^{I}} g_{i} (p^{(g)}_{0} + {\mathbf{x}_{C}}_{i}^{T} \mathbf{W}_{C} \mathbf{p}^{(g)}) \\&&\!- \log(1 + e^{(p^{(g)}_{0} + {\mathbf{x}_{C}}_{i}^{T} \mathbf{W}_{C} \mathbf{p}^{(g)})}) \end{array} $$

Quadratic approximation of Eq. 15 given the current estimates of the parameters is as the following.

16 $$  LQ_{logr}(\mathbf{W}_{C}, \mathbf{p}^{(g)}, p^{(g)}_{0}) = \frac{1}{2} {\sum\limits_{i}^{I}} q_{i} (z_{i} - p^{(g)}_{0} - {\mathbf{x}_{C}}_{i}^{T} \mathbf{W}_{C} \mathbf{p}^{(g)})^{2} $$

where

17 $$ \begin{array}{@{}rcl@{}} q_{i} & =& \tilde{p_{i}} (1 - \tilde{p_{i}}) \\ z_{i} & = &\tilde{p}^{(g)}_{0} + {\mathbf{x}_{C}}_{i}^{T} \tilde{\mathbf{W}}_{C} \tilde{\mathbf{p}}^{(g)} + \frac{g_{i} - \tilde{p_{i}}}{\tilde{p_{i}} (1 + \tilde{p_{i}})} \\ \tilde{p_{i}} & =& e^{(\tilde{p^{(g)}_{0}} + {\mathbf{x}_{C}}_{i}^{T} \tilde{\mathbf{W}_{C}} \tilde{\mathbf{p}^{(g)}})} / (1 + e^{(\tilde{p^{(g)}_{0}} + {\mathbf{x}_{C}}_{i}^{T} \tilde{\mathbf{W}_{C}} \tilde{\mathbf{p}^{(g)}})}) \end{array} $$

The parameters denoted with the $\tilde {}$ symbol are the current parameters. With the quadratic approximation now replacing the negative log-likelihood in Eq. 7 and the rescaled weighting parameter *β* used instead of *α* (see Eq. 8), the objective function becomes:

18 $$ \begin{array}{@{}rcl@{}} &&L(\mathbf{W}_{C}, \mathbf{P}^{(X)}_{C}, \mathbf{p}^{(g)}, p^{(g)}_{0})\\ & =& \frac{\beta}{2} {\sum\limits_{i}^{I}} q_{i} (z_{i} - p^{(g)}_{0} - {\mathbf{x}_{C}}_{i}^{T} \mathbf{W}_{C} \mathbf{p}^{(g)})^{2} \\ && + (1 - {\beta}) {\sum\limits_{i}^{I}} \left\| {\mathbf{x}_{C}}_{i} - {\mathbf{x}_{C}}_{i}^{T} \mathbf{W}_{C} (\mathbf{P}_{C}^{(X)})^{T} \right\|^{2}_{2} \\ && + {\sum\limits^{R}_{r}} {\lambda_{L}}_{r} \left| {\mathbf{w}_{C}}_{r} \right|_{1} + {\sum\limits^{R}_{r}} {\sum\limits^{K}_{k}} {\lambda_{G}}_{r} \sqrt{J_{k}} \left\| \mathbf{w}^{(k)}_{r} \right\|_{2} \\&&+ \lambda_{R} \left\| \mathbf{p}^{(g)} \right\|^{2}_{2} \end{array} $$

where *q*_*i*_ and *z*_*i*_ are defined as in Eq. 17. The optimization problem in Eq. 18 can be solved with an alternating procedure where the loadings $\mathbf {P}^{(X)}_{C}$ and the regression coefficients $\mathbf {p}^{(g)}, p^{(g)}_{0}$ are solved for conditional upon fixed values for the weights **W**_*C*_ and vice versa. The sparse group lasso problem for **W**_*C*_ is treated via coordinate descent (Friedman et al., 2010a), while closed-form solutions exist for the conditional updates of $\mathbf {p}^{(g)}, p^{(g)}_{0}$ and $\mathbf {P}_{C}^{(X)}$. The derivation of these updating rules can be found in Appendices C and D. After each run of conditional estimation of the parameters, the quadratic approximation in Eq. 18 is updated with new values of *q*_*i*_ and *z*_*i*_ calculated with the current parameters. To prevent the divergence of the coefficients, when the absolute difference between the current probability $\tilde {p}_{i}$ and 1 is less or equal to 10^− 5^, $\tilde {p}_{i}$ is fixed at 1. This follows the recommendation of Friedman et al., (2010b) which proposed a framework of combining regularization with GLM.

A schematic outline of the algorithm is provided in what follows. The optimization procedure that we propose here closely follows those proposed for SCaDS and SPCovR (de Schipper and Van Deun, 2018; Van Deun et al., 2018). This procedure boils down to solving for all components together (unlike deflation methods that solve for each component in turn). The alternating routine continues until the algorithm converges to a stationary point, usually a local minimum. To avoid local minima problems, we recommend using multiple random and a rational starting value based on PCovR.

## Appendix C: Estimation of **W**_*C*_

Conditional estimation of **W**_*C*_ given the other parameters **P**^(*X*)^, **p**^(*g*)^ and $p^{(g)}_{0}$ pertains to a sparse group lasso problem. The SCD-Cov-logR objective function with the quadratic approximation of the negative log-likelihood (Eq. 18) is first arranged with respect to the weights corresponding to predictor block *k* and component *r*^∗^:

19 $$ \begin{array}{@{}rcl@{}} && L(\mathbf{w}^{(k)}_{r^{*}}, \mathbf{P}^{(X)}_{C}, \mathbf{p}^{(g)}, p^{(g)}_{0}) =\\ && \frac{{\beta}}{2} \sum\limits_{i}^{{I}} q_{i} \left( z_{i} - p^{(g)}_{0} - {\sum\limits_{r}^{R}} \sum\limits_{l \neq k}^{K} p^{(g)}_{r} {\mathbf{x}^{(l)}_{i}}^{T} \mathbf{w}^{(l)}_{r}\right.\\ &&{\kern42pt}\left. - \sum\limits_{r \neq r^{*}}^{R} p^{(g)}_{r} {\mathbf{x}^{(k)}_{i}}^{T} \mathbf{w}^{(k)}_{r} - p^{(g)}_{r^{*}} {\mathbf{x}^{(k)}_{i}}^{T} \mathbf{w}^{(k)}_{r^{*}}\right)^{2}\\ && + (1 - {\beta}) \sum\limits_{i}^{{I}} \left\| {\mathbf{x}_{C}}_{i} - {\sum\limits_{r}^{R}} \sum\limits_{l \neq k}^{K} {\mathbf{w}^{(l)}_{r}}^{T} \mathbf{x}^{(l)}_{i} {\mathbf{p}_{C}}_{r}^{(X)}\right.\\ &&{\kern53pt}\left. - \sum\limits_{r \neq r^{*}} {\mathbf{w}^{(k)}_{r}}^{T} \mathbf{x}^{(k)}_{i} {\mathbf{p}_{C}}_{r}^{(X)} - {\mathbf{w}^{(k)}_{r^{*}}}^{T} \mathbf{x}^{(k)}_{i} {\mathbf{p}_{C}}_{r^{*}}^{(X)} \right\|^{2}_{2} \\ && + \lambda_{L} \left| \mathbf{w}^{(k)}_{r^{*}} \right|_{1} + \lambda_{G} \sqrt{J_{k}} \left\| \mathbf{w}^{(k)}_{r^{*}} \right\|_{2} \end{array} $$

Taking the derivative with respect to $\mathbf {w}^{(k)}_{r^{*}}$ we get:

20 $$ \begin{array}{@{}rcl@{}} & -& {\beta} \sum\limits_{i}^{{I}} q_{i} p^{(g)}_{r^{*}} (Z^{(k)}_{i} - p^{(g)}_{r^{*}} {\mathbf{x}^{(k)}_{i}}^{T} \mathbf{w}^{(k)}_{r^{*}}) \mathbf{x}^{(k)}_{i} \\ &-& 2(1 - {\beta}) \sum\limits_{i}^{{I}} (Y^{(k)}_{i} - {\mathbf{w}^{(k)}_{r^{*}}}^{T} \mathbf{x}^{(k)}_{i}) \mathbf{x}^{(k)}_{i} \\ & + &\lambda_{L} \partial \left| \mathbf{w}^{(k)}_{r^{*}} \right|_{1} + \lambda_{G} \sqrt{J_{k}} \partial \left\| \mathbf{w}^{(k)}_{r^{*}} \right\|_{2} \end{array} $$

where

21 $$ \begin{array}{@{}rcl@{}} Z^{(k)}_{i} & =& z_{i} - p^{(g)}_{0} - {\sum\limits_{r}^{R}} \sum\limits_{l \neq k}^{K} p^{(g)}_{r} {\mathbf{x}^{(l)}_{i}}^{T} \mathbf{w}^{(l)}_{r} \\&&- \sum\limits_{r \neq r^{*}}^{R} p^{(g)}_{r} {\mathbf{x}^{(k)}_{i}}^{T} \mathbf{w}^{(k)}_{r} \\ Y_{i}^{(k)} & =& {\mathbf{x}_{C}}_{i}^{T} {\mathbf{p}_{C}}_{r^{*}}^{(X)} - \sum\limits_{l \neq k}^{K} {\mathbf{w}^{(l)}_{r^{*}}}^{T} \mathbf{x}^{(l)}_{i} \end{array} $$

The subdifferential of $\left \| \mathbf {w}^{(k)}_{r^{*}} \right \|_{2}$ is defined as the following:

22 $$  \partial \left\| \mathbf{w}^{(k)}_{r^{*}} \right\|_{2} = \begin{cases} \frac{\hat{\mathbf{w}}^{(k)}_{r^{*}}}{\left\| \hat{\mathbf{w}}^{(k)}_{r^{*}} \right\|_{2}}, & \text{if } \hat{\mathbf{w}}^{(k)}_{r^{*}} \neq \mathbf{0} \\ \in \left\{ \mathbf{u}: \left\| \mathbf{u} \right\|_{2} \leq 1 \right\} , & \text{if } \hat{\mathbf{w}}^{(k)}_{r^{*}} = \mathbf{0} \end{cases} $$

where **u** is a vector of equal length as $\mathbf {w}^{(k)}_{r^{*}}$.

The *j* th element of the subdifferential of $\partial \left | \mathbf {w}^{(k)}_{r^{*}} \right |_{1}$ is defined as the following:

23 $$ \partial \left( \left| \mathbf{w}^{(k)}_{r^{*}} \right|_{1} \right)_{j} = \begin{cases} \text{sign} \left( \hat{w}^{(k)}_{j r^{*}} \right), & \text{if } \hat{w}^{(k)}_{j r^{*}} \neq 0 \\ \in \left\{ v: \left| v \right| \leq 1 \right\} , & \text{if } \hat{w}^{(k)}_{j r^{*}} = 0 \end{cases} $$

where *v* is a scalar.

By equating Eq. 20 to zero and rearranging, the condition that an optimal solution satisfies with $\hat {\mathbf {w}}^{(k)}_{r^{*}} = \mathbf {0}$ is the following:

24 $$ \begin{array}{@{}rcl@{}} &&\left\| S \left( \sum\limits_{i}^{{I}} \left( {\beta} q_{i} p^{(g)}_{r^{*}} Z^{(k)}_{i} + 2(1 - {\beta}) Y^{(k)}_{i}\right) \mathbf{x}^{(k)}_{i}, \lambda_{L}\right) \right\|_{2} \\&&\leq \lambda_{G} \sqrt{J_{k}} \end{array} $$

where S(.) is a element-wise soft-thresholding operator.

In the case that Eq. 24 is not satisfied and thus $\hat {\mathbf {w}}^{(k)}_{r^{*}} \neq \mathbf {0} $, we find the conditions for an optimal solution for the *h* th element of the weights concerning predictor block *k* and component *r*^∗^; $w^{(k)}_{h r^{*}}$. We first write the objective function with respect to $w^{(k)}_{h r^{*}}$.

25 $$ \begin{array}{@{}rcl@{}} && L(w^{(k)}_{h r^{*}}, \mathbf{P}^{(X)}_{C}, \mathbf{p}^{(g)}, p^{(g)}_{0}) \\ &&= \frac{{\beta}}{2} \sum\limits_{i}^{{I}} q_{i} (z_{i} - p^{(g)}_{0} - {\sum\limits_{r}^{R}} {\sum\limits_{l}^{K}} \sum\limits_{j \neq h}^{J_{k}} p^{(g)}_{r} x^{(l)}_{ij} w^{(l)}_{jr} \\&&- \sum\limits_{r \neq r^{*}}^{R} \sum\limits_{l \neq k}^{K} p^{(g)}_{r} x^{(l)}_{ih} w^{(l)}_{hr} - p^{(g)}_{r^{*}} x^{(k)}_{ih} w^{(k)}_{h r^{*}})^{2}\\ && + (1 - {\beta}) \sum\limits_{i}^{{I}} \left\| {\mathbf{x}_{C}}_{i} - {\sum\limits_{r}^{R}} {\sum\limits_{l}^{K}} \sum\limits_{j \neq h}^{J_{k}} \mathbf{p}^{(X)}_{r} x^{(l)}_{ij} w^{(l)}_{jr} \right.\\&&\left.- \sum\limits_{r \neq r^{*}}^{R} \sum\limits_{l \neq k}^{K} \mathbf{p}^{(X)}_{r} x^{(l)}_{ih} w^{(l)}_{hr} - \mathbf{p}^{(X)}_{r^{*}} x^{(k)}_{ih} w^{(k)}_{h r^{*}} \right\|^{2}_{2} \\ && + \lambda_{L} \left| w^{(k)}_{h r^{*}} \right| + \lambda_{G} \sqrt{J_{k}} \left\| \mathbf{w}^{(k)}_{r^{*}} \right\|_{2} \end{array} $$

Taking the derivative with respect to $w^{(k)}_{h r^{*}}$:

26 $$ \begin{array}{@{}rcl@{}} && - {\beta} \sum\limits_{i}^{{I}} q_{i} p^{(g)}_{r^{*}} x^{(k)}_{ih} (Z_{i} - p^{(g)}_{r^{*}} x^{(k)}_{ih} w^{(k)}_{h r^{*}}) \\&&- 2(1 - {\beta}) {\sum}_{i}^{{I}} x^{(k)}_{ih} (Y_{i} - x^{(k)}_{ih} w^{(k)}_{h r^{*}}) \\ && + \lambda_{L} \partial \left| w^{(k)}_{h r^{*}} \right| + \lambda_{G} \sqrt{J_{k}} \partial \left\| \mathbf{w}^{(k)}_{r^{*}} \right\|_{2} \end{array} $$

where

27 $$ \begin{array}{@{}rcl@{}} Z_{i} & =& z_{i} - p^{(g)}_{0} - {\sum\limits_{r}^{R}} {\sum\limits_{l}^{K}} \sum\limits_{j \neq h}^{J_{k}} p^{(g)}_{r} x^{(l)}_{ij} w^{(l)}_{jr} \\&&- \sum\limits_{r \neq r^{*}}^{R} \sum\limits_{l \neq k}^{K} p^{(g)}_{r} x^{(l)}_{ih} w^{(l)}_{hr} \\ Y_{i} & =& {\mathbf{x}_{C}}^{T}_{i} {\mathbf{p}_{C}}_{r^{*}}^{(X)} - {\sum\limits_{l}^{K}} \sum\limits_{j \neq h}^{J_{k}} x^{(l)}_{ij} w^{(l)}_{j r^{*}} - \sum\limits_{l \neq k}^{K} x^{(l)}_{ih} w^{(l)}_{h r^{*}} \end{array} $$

The subdifferential of $\left \| \mathbf {w}^{(k)}_{r^{*}} \right \|_{2}$ with respect to $w^{(k)}_{h r^{*}}$ is provided in Eq. 22; it is the *h* th element of $\frac {\hat {\mathbf {w}}^{(k)}_{r^{*}}}{\left \| \hat {\mathbf {w}}^{(k)}_{r^{*}} \right \|_{2}}$. The subdifferential of $\partial \left | w^{(k)}_{h r^{*}} \right |$ is defined as the following:

28 $$ \partial \left| w^{(k)}_{h r^{*}} \right| = \begin{cases} \text{sign}\left( \hat{w}^{(k)}_{h r^{*}} \right), & \text{if } \hat{w}^{(k)}_{h r^{*}} \neq 0 \\ \in \left\{ v: \left| v \right| \leq 1 \right\} , & \text{if } \hat{w}^{(k)}_{h r^{*}} = 0 \end{cases} $$

where *v* is a scalar.

We can equate the derivate to zero to find the optimality conditions for $\hat {w}^{(k)}_{h r^{*}}$, which can be summarized by the following:

29 $$  \hat{w}^{(k)}_{h r^{*}} = \frac{S({\sum}_{i}^{{I}} x^{(k)}_{ih} ({\beta} p^{(g)}_{r^{*}} q_{i} Z_{i} + 2(1 - {\beta}) Y_{i}), \lambda_{L})}{{\beta} {p^{(g)}_{r^{*}}}^{2} {\sum}_{i}^{{I}} q_{i} {x^{(k)}_{ih}}^{2} + 2(1 - {\beta}) {\sum}_{i}^{{I}} {x^{(k)}_{ih}}^{2} + \lambda_{G} \sqrt{J_{k}} / \left\| \mathbf{w}^{(k)}_{r^{*}} \right\|_{2}} $$

With these conditions, we can set up the following coordinate descent algorithm.

## Appendix D: Estimation of $\mathbf {p}^{(g)}, p^{(g)}_{0}$ and $\mathbf {P}_{C}^{(X)}$

Closed-form solutions exist for the regression coefficients and the intercept.

30 $$ \begin{array}{@{}rcl@{}} \hat{\mathbf{p}}^{(g)} &=& [ {(\mathbf{X}_{C} \mathbf{W}_{C})}^{T} \mathbf{Q} \mathbf{X}_{C} \mathbf{W}_{C} + (2/ \alpha) \lambda_{R} \mathbf{I}_{R} ]^{-1} \\&&[{(\mathbf{X}_{C} \mathbf{W}_{C})}^{T} \mathbf{Q} \mathbf{z} - p^{(g)}_{0} {(\mathbf{X}_{C} \mathbf{W}_{C})}^{T} \mathbf{q} ] \end{array} $$

31 $$ \hat{p}^{(g)}_{0} = \left( {\sum}_{i}^{{I}} q_{i} \left( z_{i} - {\mathbf{x}_{C}}_{i}^{T} \mathbf{W}_{C} \mathbf{p}^{(g)}\right)\right) / \left( {\sum}_{i}^{{I}} q_{i}\right) $$

where **Q** is a diagonal matrix with the *i* th diagonal element being *q*_*i*_. **q** and **z** are vectors with the elements being *q*_*i*_ and *z*_*i*_ respectively, which are defined in Eq. 17.

The loadings $\mathbf {P}^{(X)}_{C}$ are also obtained via a closed-form solution; $\mathbf {P}^{(X)}_{C} = \mathbf {U} \mathbf {V}^{T}$ where **U** and **V** are found through singular value decomposition of $\mathbf {X}^{T}_{C} \mathbf {X}_{C} \mathbf {W}_{C} = \mathbf {U} \mathbf {D} \mathbf {V}^{T}$.

## Appendix E: SCD-Cov-logR multiclass algorithm

Like for the binary problem, the solution to Eq. 10 is found by iteratively reweighted least squares. Partial quadratic approximation can be conducted such that only parameters that concern the *m* th category can vary at a time. With the quadratic approximation replacing the negative log-likelihood in Eq. 10 and the rescaled weighting parameter *β* used instead of *α* (see Eq. 8), the objective function becomes:

32 $$ \begin{array}{@{}rcl@{}} &&L(\mathbf{W}_{C}, \mathbf{P}^{(X)}_{C}, \mathbf{p}_{m}^{(g)}, {p_{0}}^{(g)}_{m})\\ &= & \frac{\beta}{2} {{\sum}^{I}_{i}} q_{i} (z_{i} - {p_{0}}^{(g)}_{m} - {\mathbf{x}_{C}}_{i}^{T} \mathbf{W}_{C} \mathbf{p}_{m}^{(g)})^{2} \\ & &+ (1 - \beta) {{\sum}^{I}_{i}} \left\| {\mathbf{x}_{C}}_{i} - {\mathbf{x}_{C}}_{i}^{T} \mathbf{W}_{C} (\mathbf{P}_{C}^{(X)})^{T} \right\|^{2}_{2} \\ && + {\sum\limits_{r}^{R}} {\lambda_{L}}_{r} \left| {\mathbf{w}_{C}}_{r} \right|_{1} + {\sum\limits_{r}^{R}} {{\sum}_{k}^{K}} {\lambda_{G}}_{r} \sqrt{J_{k}} \left\| \mathbf{w}^{(k)}_{r} \right\|_{2} \\&&+ \lambda_{R} \left\| \mathbf{p}_{m}^{(g)} \right\|^{2}_{2} \end{array} $$

where

33 $$ \begin{array}{@{}rcl@{}} q_{i} & =& \tilde{p_{i}} (1 - \tilde{p_{i}}) \\ z_{i} & = &\tilde{p_{0}}^{(g)}_{m} + {\mathbf{x}_{C}}_{i}^{T} \tilde{\mathbf{W}}_{C} \tilde{\mathbf{p}}_{m}^{(g)} + \frac{g_{i} - \tilde{p_{i}}}{\tilde{p_{i}} (1 + \tilde{p_{i}})} \\ \tilde{p_{i}} & =& e^{(\tilde{p_{0}}^{(g)}_{m} + {\mathbf{x}_{C}}_{i}^{T} \tilde{\mathbf{W}_{C}} \tilde{\mathbf{p}}_{m}^{(g)})} / (1 + {\sum}^{M-1}_{m} e^{(\tilde{p_{0}}^{(g)}_{m} + {\mathbf{x}_{C}}_{i}^{T} \tilde{\mathbf{W}_{C}} \tilde{\mathbf{p}}_{m}^{(g)})}) \end{array} $$

the parameters denoted with the $\tilde {}$ symbol are the current parameters. The loadings are constrained to be column-orthogonal: $(\mathbf {P}_{C}^{(X)})^{T} \mathbf {P}_{C}^{(X)} = \mathbf {I}_{R}$. This optimization problem can be solved with an alternating procedure similar to that of the binary classification. In fact, the conditional estimation of the parameters is done in the same way as for the binary problem (shown in Appendices C and D) with a small tweak on the definition of certain quantities. We can first notice that this objective function with quadratic approximation with respect to category *m* can be considered as a binary problem between category *m* and the baseline category *M*. It can be seen that the only difference between the functions for the multiclass (Eqs. 32 and 33) and the binary (Eqs. 18 and 17) problems is the definition of the current parameter $\tilde {p_{i}}$. Therefore, from the binary objective function (Eq. 18), computing $\tilde {p_{i}}$ by following (Eq. 33) and replacing the regression coefficients $\mathbf {p}^{(g)}, p^{(g)}_{0}$ into $\mathbf {p}_{m}^{(g)}, {p_{0}}^{(g)}_{m}$ specific for category *m* would enable us to rely on the same solutions for the conditional updates of the quantities $\mathbf {W}_{C}, \mathbf {p}_{m}^{(g)}, {p_{0}}^{(g)}_{m}$ and $\mathbf {P}_{C}^{(X)}$. The algorithm for the multiclass problem however cycles over the *M* − 1 categories on top of the conditional updates of the quantities. After each run of conditional estimation of the quantities, the quadratic approximation in Eq. 32 is updated with new values of *q*_*i*_ and *z*_*i*_ calculated with the current parameters.

A schematic outline of the algorithm is provided below. The alternating routine continues until the algorithm converges to a stationary point, usually a local minimum. To avoid local minima problems, we recommend using multiple random and a rational starting value based on PCovR.

## Appendix G: Toy example dataset: model selection via exhaustive grid search of all parameters

Instead of the sequential model selection procedure adopted in the toy example dataset (“Toy example”), we have conducted cross-validation (CV) in which all of the possible parameters are crossed exhaustively. The ranges of the parameters considered were the same as in the sequential procedure:

- *β*: [0.1,0.2,0.3,0.4,0.5,0.6,0.7,0.8,0.9]
- *λ*_*R*_: [0.1,0.5,1,3,5,10,30,50]
- *λ*_*L*_: [0.5,1,5,7,10,15,30,45,100]
- *λ*_*G*_: [0.1,0.5,1,2,5,10]

For the number of covariates R, we adopted the range of [1,2,3,4] because PCA on the predictor data matrix revealed that from the fifth component onwards, the proportion of explained variance is smaller than 5% (this has been depicted in Appendix F). Crossing all of the possible parameters, we administered fivefold CV to 15552 models in total.

The model with the smallest CV error was characterized by the parameters: *R* = 3,*β* = 0.6,*λ*_*R*_ = 0.5,*λ*_*L*_ = 10, *λ*_*G*_ = 5. The estimated weights and regression coefficients are reported in Table 7. It can be seen that the estimates are very similar to the ones found by the model obtained through the sequential approach of CV.

**Table 7:** Weights and regression coefficients provided by the three-covariate model with the smallest cross-validation error

Weights				Logistic regression coefficients	
Block 1					
x1	0.420	0	0	1	-1.272
x2	0.420	0	0	2	-0.096
x3	0.439	0	0	3	1.499
x4	0.486	0	0	intercept	-0.206
x5	0	0	0.330		
x6	0	0	0.324		
x7	0	0	0.288		
x8	0	0	0.261		
x9	0	0	0		
x10	0	0	0		
x11	0	0	0		
x12	0	0	0		
x13	0	0	0		
x14	0	0	-0.021		
x15	0	0	0		
Block 2					
x16	0	0	0.343		
x17	0	0	0.361		
x18	0	0	0.316		
x19	0	0	0.256		
x20	0	0.437	0		
x21	0	0.429	0		
x22	0	0.439	0		
x23	0	0.470	0		
x24	0	0	0		
x25	0	0	0		
x26	0	-0.085	0		
x27	0	0	0		
x28	0	0	0		
x29	0	0	0		
x30	0	0	0		

If we apply the one standard error rule to select the simplest model among those within 1 SE from the minimum CV error, we would need to make a choice regarding which parameter to look consider first. The number of covariates *R* can be considered as the most influential parameter, followed by the weighting parameter *β*. Prioritizing these two parameters, the one standard error rule selects the model: *R* = 2,*β* = 0.5,*λ*_*R*_ = 0.5,*λ*_*L*_ = 30,*λ*_*G*_ = 1. Table 8 shows the estimates of this two-covariate model. It can be seen that the covariate which is distinctive to the second predictor block (D2 in Table 1) is excluded from this model. This is sensible because this covariate was defined to have a very small predictive influence on the outcome variable when generating the data: population value of the logistic regression weight was set at -0.01. Hence, it is natural that the exhaustive CV approach that only considers the prediction error could result in omitting this covariate. The two covariates extracted are in agreement to the covariates found by the sequential approach of CV.

**Table 8:** Weights and regression coefficients provided by the two-covariate model with the one standard error rule

Weights			Logistic regression coefficients	
Block 1			1	− 1.263
x1	0.369	0	2	1.481
x2	0.380	0	intercept	− 0.199
x3	0.507	0		
x4	0.427	0		
x5	0	0.345		
x6	0	0.311		
x7	0	0.265		
x8	0	0.212		
x9	0	0		
x10	0	0		
x11	0	0		
x12	0	0		
x13	0	0		
x14	0	0		
x15	0	0		
Block 2				
x16	0	0.337		
x17	0	0.378		
x18	0	0.302		
x19	0	0.206		
x20	0	0		
x21	0	0		
x22	0	0		
x23	0	0		
x24	0	0		
x25	0	0		
x26	0	0		
x27	0	0		
x28	0	0		
x29	0	0		
x30	0	0		

## Appendix H: Data generation for multiclass toy example dataset

The data generating setup employed for our simulation study is adapted slightly such that it can generate more than two categories, in generating the toy example dataset for the multiclass classification problem. As for the simulation study, two blocks of predictor variables were generated from three underlying covariates; one distinctive covariate per each predictor block and one common covariate. Each predictor block comprised of 15 variables (*J* = 30 in total), and *I* = 1000 observation units were generated. With the population weights and logistic regression coefficients provided in Table 4, the toy example dataset was generated via the following setup:

$$ \begin{array}{@{}rcl@{}} && \mathbf{T} \sim \mathcal{M}\mathcal{V}\mathcal{N}(\mathbf{0}, \mathbf{\Sigma} = 50^{2} \mathbf{I}_{3}) \\ && \mathbf{E} \sim \mathcal{M}\mathcal{V}\mathcal{N}(\mathbf{0}, \mathbf{\Sigma}_{E} = \sigma^{2} \mathbf{I}_{J}) \\ && \mathbf{X}_{C} \gets \mathbf{T} \mathbf{W}_{C}^{T} + \mathbf{E} \end{array} $$

34 $$ \begin{array}{@{}rcl@{}} && \mathbf{z}_{m} \gets exp(\mathbf{T} \mathbf{p}_{m}^{(g)}) / (1 + exp({\sum}_{m'}^{M-1}\mathbf{T} \mathbf{p}_{m^{\prime}}^{(g)}))\\ && \text{ for } m = 1, \ldots, M-1 \\ && \mathbf{z}_{M} \gets 1 / (1 + exp({\sum}_{m^{\prime}}^{M-1}\mathbf{T} \mathbf{p}_{m^{\prime}}^{(g)})) \\ && g_{im} \sim Multinoulli(z_{im}) \text{ for } m = 1, \ldots, M \end{array} $$

where **T**,Σ and **W**_*C*_ are all defined in the same manner as in the simulation study (see “Design and procedure”). The predictors **X**_*C*_ are generated by multiplying the covariate scores matrix with the weights matrix and adding random error. The diagonal covariance matrix Σ_*E*_ that governs the variance of error variables **E** is specified such that the covariates **T** account for 50% of variance in **X**_*C*_. $\mathbf {p}_{m}^{(g)}$ indicates the logistic regression coefficients for the log-odds of the *m* th category as opposed to the baseline category *M* = 3. The statements in the fourth and the fifth lines together specify the (*I* = 1000 × *M* = 3) matrix **Z**; *z*_*i**m*_ denotes the probability of the *i* th observation belonging to *m* th category, defined according to the baseline-category logit model (Agresti, 2003). *g*_*i**m*_ is therefore sampled from a Multinoulli distribution defined by the prescribed probabilities *z*_*i**m*_.

## Appendix I

**Table 9:** Lasso and Group lasso penalty parameters initially fixed in the simulation study, per each condition

Dimensions	Relevant	VAF	*λ*_*G*__1_	*λ*_*G*__2_	*λ*_*G*__3_	*λ*_*L*__1_	*λ*_*L*__2_	*λ*_*L*__3_
low	D1,D2	0.8	0.5	0.5	0.5	20	10	20
low	D1,D2	0.5	0.5	0.5	0.5	30	15	30
low	D1,D2	0.2	0.5	0.5	0.5	30	15	30
low	D1,C	0.8	0.5	0.5	0.5	30	15	30
low	D1,C	0.5	0.5	0.5	0.5	30	15	30
low	D1,C	0.2	0.5	0.5	0.5	30	15	30
high	D1,D2	0.8	3	3	3	15	7.5	15
high	D1,D2	0.5	2	2	2	30	15	30
high	D1,D2	0.2	1	1	1	20	10	20
high	D1,C	0.8	1	1	1	10	10	10
high	D1,C	0.5	1	1	1	30	15	30
high	D1,C	0.2	1	1	1	10	10	10

## Appendix K: Models constructed from the 500 Family dataset using the related methods

**Table 10:** Estimates provided by PCR (SCaDS-logR), DIABLO and regularized logistic regression for the 500 Family dataset

	SCaDS-logR		DIABLO		LogR
	1	2	1	2	b
Mother					
Relationship with partners	0	0.243	0	0	0
Argue with partners	0	0.247	0	0	0
Child’s bright future	0	0	0	0	0
Activities with children	0	0	0	0	0
Feeling about parenting	0	0.175	0	0	0
Comunication with children	0	0.338	0	0	0
Argue with children	0	0.152	0	0	0
Confidence about oneself	0	0.382	0	0	0
Father					
Relationship with partners	0	0.097	0	0	0
Argue with partners	0	0.208	0	0	0
Child’s bright future	0	0	0	1	0.058
Activities with children	0	0	0	0	0
Feeling about parenting	0	0	0	0	0
Comunication with children	0	0	0	0	0
Argue with children	0	0.255	0	0	0
Confidence about oneself	0	0.047	0	0	0
Child					
Child self-confidence/esteem	0.274	0	0	0	0
Social life and extracurricular	0.333	0	0	0	0
Importance of friendship	0.460	0	0	0	0
Self-image	0.360	0	1	0	0.278
Happiness	0.371	0	0	0	0
Confidence about the future	0.275	0	0	0	0

## Footnotes

1 $\hat {y}_{i} = {\sum }_{r} p_{r}^{(y)} t_{ir} = {\sum }_{r} ({\sum }_{j} p_{r}^{(y)} x_{i j} w_{j r}) = {\sum }_{j} ({\sum }_{r} p_{r}^{(y)} w_{j r}) x_{i j}$, with ${\sum }_{r} p_{r}^{(y)} w_{j r}$ as a regression coefficient for the *j* th predictor, where *r* is an index for each covariate.

2 This ratio of negative log-likelihoods is used in computation of McFadden’s pseudo *R*^2^ (McFadden & et al. 1973) that provides insight on explained variance in the context of logistic regression.

3 This would then be a single model of sPLS-DA (sparse partial least squares discriminant analysis).

## Code Availability

The implementation of SCD-Cov-logR was done in R and Rcpp, which can be found on GitHub: https://github.com/soogs/SCD-Cov-logR.
